# Automated assessment of right ventricular systolic function from coronary angiograms with video-based artificial intelligence algorithms: development, validation, comparison against humans, and prospective deployment

**DOI:** 10.1093/ehjdh/ztag059

**Published:** 2026-04-15

**Authors:** Fatima Zahra Fawzi, Istok Menkovic, Nicolas Dostie, Maxime Tremblay-Gravel, Marie-Claude Parent, Gabriel Asslo, Jean-François Tanguay, Guillaume Marquis-Gravel, Minhaj Ansari, Joshua P Barrios, Geoffrey H Tison, Jacques Delfrate, Robert Avram

**Affiliations:** Division of Cardiology, Department of Medicine, Montreal Heart Institute, 5000 Belanger Street, Montreal, QC H1T 1C8, Canada; Department of Bioinformatics, Université de Montréal, 2900 Edouard Montpetit Blvd, Montreal, Quebec H3T 1J4, Canada; Department of Medicine, Université de Montréal, 2900 Edouard Montpetit Blvd, Montreal, Quebec H3T 1J4, Canada; Department of Medicine, Université de Montréal, 2900 Edouard Montpetit Blvd, Montreal, Quebec H3T 1J4, Canada; Division of Cardiology, Department of Medicine, Montreal Heart Institute, 5000 Belanger Street, Montreal, QC H1T 1C8, Canada; Department of Medicine, Université de Montréal, 2900 Edouard Montpetit Blvd, Montreal, Quebec H3T 1J4, Canada; Division of Cardiology, Department of Medicine, Montreal Heart Institute, 5000 Belanger Street, Montreal, QC H1T 1C8, Canada; Department of Medicine, Université de Montréal, 2900 Edouard Montpetit Blvd, Montreal, Quebec H3T 1J4, Canada; Division of Cardiology, Department of Medicine, Montreal Heart Institute, 5000 Belanger Street, Montreal, QC H1T 1C8, Canada; Department of Bioinformatics, Université de Montréal, 2900 Edouard Montpetit Blvd, Montreal, Quebec H3T 1J4, Canada; Division of Cardiology, Department of Medicine, Montreal Heart Institute, 5000 Belanger Street, Montreal, QC H1T 1C8, Canada; Department of Medicine, Université de Montréal, 2900 Edouard Montpetit Blvd, Montreal, Quebec H3T 1J4, Canada; Division of Cardiology, Department of Medicine, Montreal Heart Institute, 5000 Belanger Street, Montreal, QC H1T 1C8, Canada; Department of Medicine, Université de Montréal, 2900 Edouard Montpetit Blvd, Montreal, Quebec H3T 1J4, Canada; Division of Cardiology, Department of Medicine, University of California, SanFrancisco, Cardiology (San Francisco, CA, United States), 505 Parnassus Avenue, San Francisco, CA 94143, USA; Cardiovascular Research Institute, University of California, SanFrancisco, CA 94143, USA; Bakar Computational Health Sciences Institute, University of California, SanFrancisco 94158, USA; Division of Cardiology, Department of Medicine, University of California, SanFrancisco, Cardiology (San Francisco, CA, United States), 505 Parnassus Avenue, San Francisco, CA 94143, USA; Cardiovascular Research Institute, University of California, SanFrancisco, CA 94143, USA; Bakar Computational Health Sciences Institute, University of California, SanFrancisco 94158, USA; Division of Cardiology, Department of Medicine, University of California, SanFrancisco, Cardiology (San Francisco, CA, United States), 505 Parnassus Avenue, San Francisco, CA 94143, USA; Cardiovascular Research Institute, University of California, SanFrancisco, CA 94143, USA; Bakar Computational Health Sciences Institute, University of California, SanFrancisco 94158, USA; Division of Cardiology, Department of Medicine, Montreal Heart Institute, 5000 Belanger Street, Montreal, QC H1T 1C8, Canada; HeartWise.ai, 5000 Belanger Street, QC H1T 1C8, Montreal, Quebec, Canada; Division of Cardiology, Department of Medicine, Montreal Heart Institute, 5000 Belanger Street, Montreal, QC H1T 1C8, Canada; Department of Bioinformatics, Université de Montréal, 2900 Edouard Montpetit Blvd, Montreal, Quebec H3T 1J4, Canada; HeartWise.ai, 5000 Belanger Street, QC H1T 1C8, Montreal, Quebec, Canada

**Keywords:** Machine learning, Artificial intelligence, Coronary angiography, Angiogram, Automated interpretation, Right ventricular systolic function, Right ventricle, Transthoracic echocardiogram

## Abstract

**Aims:**

Right ventricular systolic function (RVSF) is a critical determinant of cardiovascular outcomes, yet assessment during coronary angiography remains challenging without prior imaging. We developed and validated DeepRV, a deep learning model predicting RVSF from routine coronary angiograms.

**Methods and results:**

DeepRV, a video-based deep neural network, was developed using 8053 coronary angiography studies from 6923 patients at Montreal Heart Institute (2017–23), with RVSF determined by echocardiography. The model was externally validated at the University of California, San Francisco, and prospectively deployed during primary percutaneous coronary intervention (PCI) for ST-segment elevation myocardial infarction (STEMI). In the internal test set (*n* = 1586; 10.5% reduced RVSF), DeepRV achieved area under the receiver operating characteristic curve (AUROC) 0.80 [95% confidence interval (CI): 0.76–0.84], sensitivity 70.5%, specificity 78.5%, and negative predictive value 95.8%. External validation demonstrated AUROC 0.75 (95% CI: 0.72–0.77) on the UCSF dataset (*n* = 2247 studies; 30% reduced RVSF). Prospective deployment of DeepRV during STEMI cases at our institution (*n* = 82) achieved AUROC 0.83 (95% CI: 0.71–0.93) using post-PCI angiogram with a median 5.1 s inference time. In a human performance evaluation (*n* = 200), artificial intelligence (AI) assistance improved accuracy of identifying RVSF for cardiologists (72.1–77.6%) and medical students (43.5–64.0%). Artificial intelligence alone achieved the highest accuracy (79.5%) and sensitivity (70.0%), while cardiologists with AI achieved the highest specificity (84.6%).

**Conclusion:**

DeepRV enables automated RVSF assessment from routine coronary angiograms and enhances diagnostic accuracy across experience levels. Real-time inference and open-weight availability support its potential as a point-of-care tool for risk stratification during coronary angiography.

## Graphical Abstract

**Graphical Abstract:**
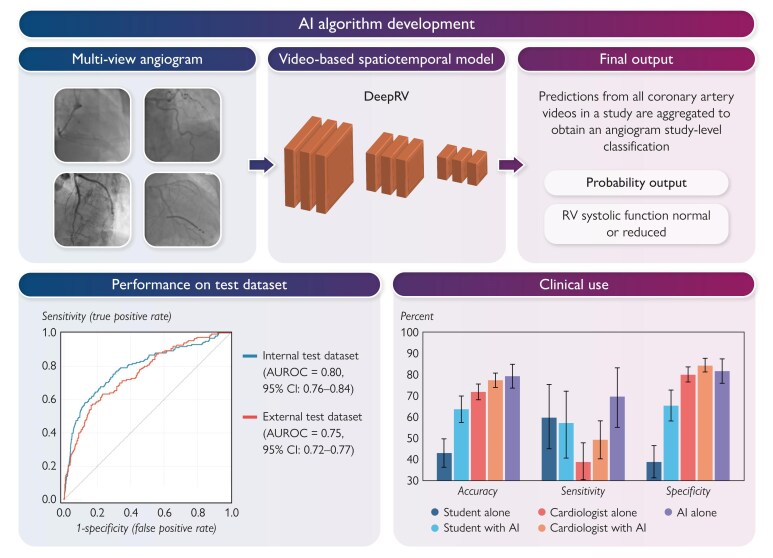

## Introduction

Right ventricular systolic function (RVSF) guides critical decisions including mechanical circulatory support selection, with strong prognostic implications.^1–6^ However, RVSF assessment remains challenging during urgent coronary angiography (CAG) when prior imaging is unavailable.^7–9^ Transthoracic echocardiography (TTE) is a non-invasive way to assess RVSF, but it is often unavailable in the cath lab during acute coronary syndrome (ACS), when rapid RVSF evaluation is crucial.^9^ Also images are of limited quality in those with poor acoustic windows.^10–12^

Coronary angiography remains the gold standard for coronary artery disease diagnosis. Using artificial intelligence (AI), we can extract digital biomarkers from these routine videos, yet RVSF prediction from angiograms remains unexplored despite the angiogram capturing real-time cardiac motion.^7,13^ We developed DeepRV, an open-weight video-based deep neural network (DNN) that predicts RVSF from routine left and right coronary angiograms and validated in an external healthcare system. We also prospectively deployed the algorithm in patients undergoing urgent CAG for ST-elevation myocardial infarction using our bespoke software in the catheterization laboratory and also compared the increment value of DeepRV against cardiologists’ interpretation of the angiogram for RVSF.

## Methods

### Study datasets and study population

We conducted a retrospective study, including patients aged 18 years or older who underwent CAG between 1 January 2017 and 31 December 2023 at the Montreal Heart Institute (MHI) and had TTE performed either up to one month prior to or within 5 days after the CAG (see Supplementary material online, *Figure S1*). The study was approved by the MHI Review Ethics Board (#2025-3459). Informed consent was waived due to the retrospective nature of the study.

Coronary angiogram videos of a minimum of 7 frames, at 7 or 15 frames per second, were obtained in DICOM format from three vendors (see Supplementary material online, *Methods*). Each CAG video paired with its corresponding TTE constituted a single observation. We identified the diagnostic phase of the CAG procedure based on time stamps and the parentheses of a guide wire. The first videos of the left coronary artery (LCA) and right coronary artery (RCA) without guide wire were considered diagnostic. Any videos that followed with the guide wire were considered interventional, and any videos of the left or right coronary after the guide wire was withdrawn were considered post–percutaneous coronary intervention (PCI). The model was trained only on diagnostic videos. To prevent data leakage and ensure independent datasets for training, development, and evaluation, we split the data at the patient level, with their respective coronary angiograms: 70% for training, 10% for development, and 20% for final model evaluation (see Supplementary material online, *Figure S2*).

### Clinical, echocardiographic, and haemodynamic variables

Clinical data included demographics (age, sex), angiographic projections (Supplemnetary material online, *Table S1*) and angiography indications categorized as ACS (STEMI, NSTEMI/unstable angina) or non-ACS conditions (arrhythmias, heart failure and cardiomyopathies, valvular and prosthetic heart disease, ischaemic heart disease, and other diagnostic indications). Right ventricular systolic function was assessed via TTE using a combination of tricuspid annular plane systolic excursion (TAPSE) (normal > 17 mm, reduced ≤ 17 mm), fractional area change (FAC), and visual assessment (eyeballing), in accordance with the 2025 ASE guidelines.^14^ Haemodynamic validation in a subset with right heart catheterization (RHC) included PAPi (<2.0 abnormal)^15,16^ and RA/PCWP ratio (≥0.63 abnormal).^17^ In our institution, the RHC is always performed before the angiogram. Missing data rows were not considered for analysis. See Supplementary material online, *Methods* for additional information.

### Algorithm development

**Figure 1:**
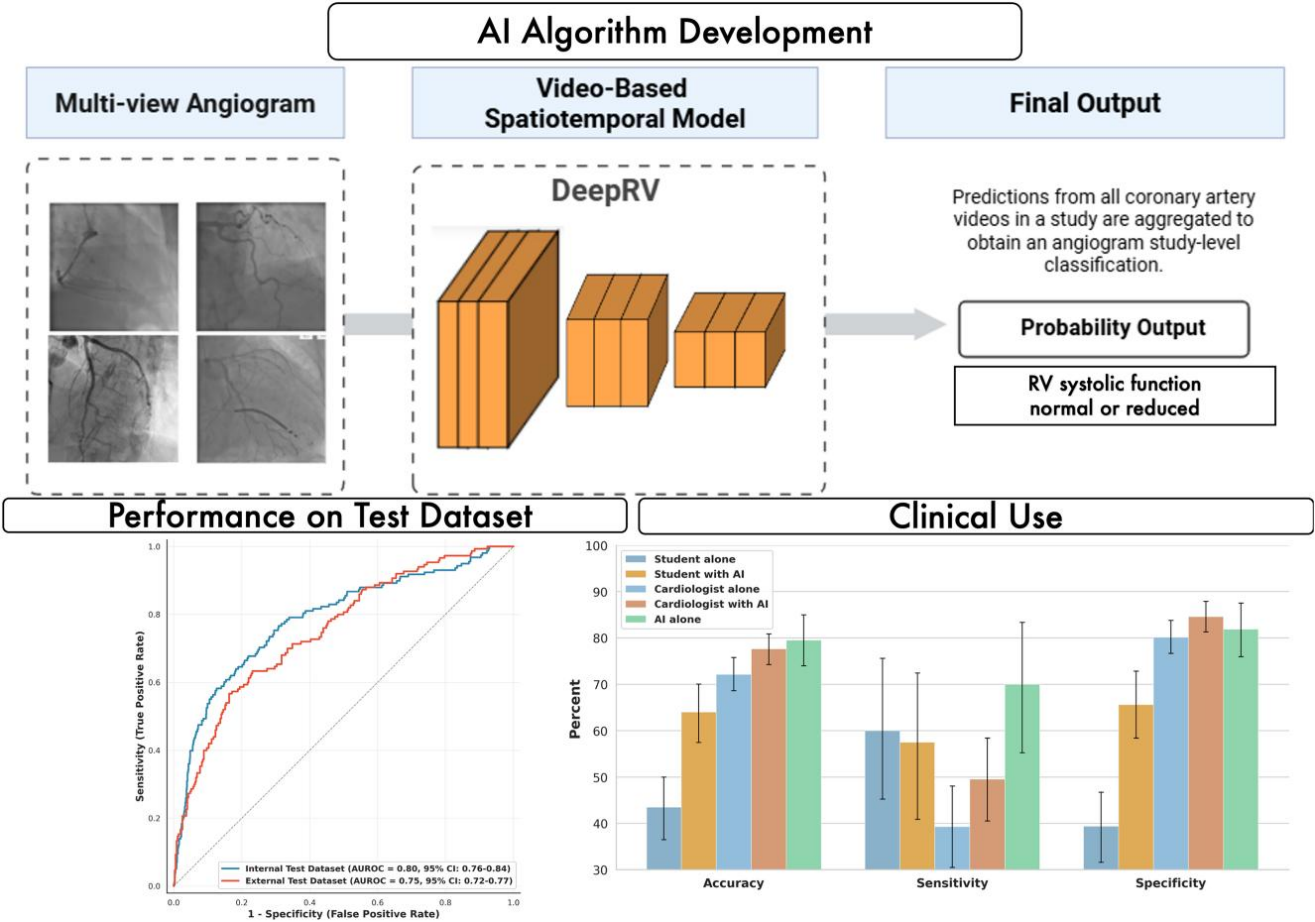
DeepRV overview. Top panel: Overview of the DeepRV pipeline. Multi-view coronary angiogram videos from left and right coronary arteries are processed through a video-based spatiotemporal deep neural network. Predictions from all videos within a study are aggregated to generate a study-level probability output classifying right ventricular systolic function as normal or reduced. Bottom left panel: Receiver operating characteristic curves demonstrating model discrimination in the internal test set (AUROC 0.80, 95% CI: 0.76–0.84; blue) and external validation cohort (AUROC 0.75, 95% CI: 0.72–0.77; orange). Bottom right panel: Human performance evaluation comparing diagnostic accuracy, sensitivity, and specificity across readers with varying experience levels (students, cardiologists) with and without AI assistance. AI alone achieved the highest accuracy (79.5%) and sensitivity (70.0%), while cardiologists with AI achieved the highest specificity (84.6%). AI assistance improved accuracy across all experience levels, with the largest gains observed in less experienced readers.

DeepRV, a video-based DNN, was built on an X3D-M architecture^18^ (and we also explored MVIT-2B-S architecture) and was trained to classify RVSF on TTE as normal or reduced from raw RCA and LCA angiogram videos (*Figure 1*). To identify the left or right CAG, we used our previously published video classifier which achieved an AUROC ≥ 0.95 for detection of these structures.^19^ DeepRV was initialized using pre-trained weights from the Kinetics400^20^ dataset to leverage transfer learning and trained to minimize binary cross-entropy loss. Optimization was performed using a stochastic gradient descent^21^ with a learning rate of 0.007.^21^ Detailed training parameters and hyperparameter optimization procedures are described in Supplementary material online, *Methods* and *Tables S2* and *S3*. To interpret our DeepRV algorithm’s decision-making process, we employed two complementary explainability techniques: Grad-CAM^22^ and Guided Backpropagation.^23^ Full details of data pre-processing, model training, evaluation protocols, visualization techniques, and statistical analyses are provided in the Supplementary material online, *Methods*.

To test whether DeepRV captures RV-specific features beyond LV-RV coupling, we applied CathEF, our previously published angiography model for the left ventricular ejection fraction (LVEF) estimation, to the DeepRV test set, averaging clip-level predictions to obtain a study-level LVEF estimate.^24^ Because lower LVEF is expected to correlate with RV dysfunction through ventricular coupling, we negated CathEF outputs so that higher values correspond to greater predicted RV dysfunction risk, aligning directionality with DeepRV output.

### External validation

To assess the generalizability of DeepRV, we conducted an external validation study using coronary angiograms from the University of California of San Francisco (UCSF). The UCSF dataset included randomly selected CAG performed between December 2012 and December 2019, each paired with a TTE conducted within 30 days prior or up to 5 days following the angiogram. Inclusion criteria were aligned with those of the MHI dataset to ensure consistency in patient selection.^19^ Subsequently, the videos were processed through two parallel pathways: the DeepRV model for RVSF assessment and a Coronary Dominance Classifier (Swin3D^25^) for determining coronary dominance patterns classified as left or right dominant.

### Prospective evaluation

We took the best-performing model and deployed it in our pictures archive communication system (PACS) using our bespoke software PACS-AI.^26^ At MHI, coronary angiogram videos flow directly into the PACS as soon as they are acquired and PACS-AI^26^ enables the application of AI models on these videos at point-of-care by the X-ray technicians during the procedure in real time. We conducted a prospective evaluation of the model by applying DeepRV by the interventional cardiologist on consecutive STEMI patients between January 2025 and June 2025, while they were undergoing primary PCI. We used as input up to four videos in the diagnostic phase (before any guidewire was inserted in a coronary artery) as well as up to four videos in the post-PCI phase (after all stents were inserted in the culprit artery) and compared the results of DeepRV using videos from either phase against ground truth obtained on TTE within 24–48 h.

### Human performance evaluation

To evaluate human diagnostic performance with and without AI assistance, we randomly sampled 200 studies from the internal held-out test set using stratified sampling to ensure clinical relevance [40 studies with RV dysfunction (20%), 160 normal studies (80%)]. For each study, left and right CAG videos were combined into a single side-by-side display when both views were available (*n* = 165 combined, *n* = 32 left-only, *n* = 3 right-only). Four readers (three board-certified interventional cardiologists and one medical student) independently reviewed each study on an annotation platform (Labelbox.com) under two conditions: (i) video alone and (ii) video with AI prediction overlay displaying a normalized risk score (0–100) and binary classification (see Supplementary material online, *Figure S1*). Studies were presented in randomized order with a minimum 1-week washout period between conditions to minimize recall bias. Readers were blinded to ground truth RV function status.

### Statistical analysis

DeepRV performance was evaluated using area under the receiving operating characteristic curve (AUROC), the area under the precision–recall curve (AUPRC), sensitivity, specificity, positive predictive value (PPV), negative predictive value (NPV), and diagnostic odds ratio (DOR)^27^ in line with the TRIPOD-AI recommendations.^28^ Study-level predictions were averaged across all videos, with optimal threshold determined by Youden’s index^29^ on the training set to maximize sensitivity and specificity. Model calibration was assessed using the estimated calibration index^30^ with spline-based recalibration. Subgroup analyses examined performance across: (i) demographics (age groups: <50, 50–64, 65–74, ≥75 years; sex); (ii) clinical presentations [ACS subtypes including STEMI stratified by culprit vessel (RCA vs. LCA), NSTEMI/unstable angina; non-ACS indications including arrhythmias, heart failure/cardiomyopathies, valvular disease, ischaemic heart disease]; (iii) cardiac conditions [presence of atrial fibrillation and severity of tricuspid regurgitation (TR) (none/mild vs. moderate/severe)]; (iv) haemodynamic parameters in patients with RHC (PAPi < 2 vs. ≥2, RA/PCWP ratio < 0.63 vs. ≥0.63, cardiac output < 4 vs. ≥4 L/min); (v) angiographic characteristics [coronary dominance pattern, vessel analysed (RCA vs. LCA)]; (vi) different RV severities (see Supplementary material online, *Figure S4*); and (vii) technical factors (imaging vendor, TTE timing relative to angiography). Subgroup analyses stratified by ACS status, culprit vessel, TTE timing, and angiographic view were pre-specified. Decision curve analysis assessed clinical utility across threshold probabilities. For the human performance evaluation, we present the results stratified by reader and by status (staff vs. medical student). To determine whether DeepRV captures RV-specific features rather than simply proxying LV dysfunction, we examined its association with LVEF from concurrent echocardiography and evaluated AUROC in patients with LVEF ≤ 40% vs. >40%. As a comparator, we applied CathEF,^24^ our previously published angiography-based LVEF predictor, to the same test set. Statistical significance was set at *P* ≤ 0.05 with 95% confidence intervals (CIs) from 1000 bootstrap iterations. For the human performance, performance was assessed using accuracy, sensitivity, and specificity, calculated for each reader-condition combination and compared against ground truth clinical outcomes.

## Results

### Clinical characteristics of the Montreal Heart Institute study cohorts

The internal study cohort included 8053 studies (6035 patients, mean age 67.4 ± 12.0 years, 71% male, Supplementary material online, *Table S4*) with 833 (10.3%) showing reduced RVSF. Out of these, 833 (10.3%) demonstrated reduced (mild, moderate, or severe) RVSF, while 7220 (89.7%) exhibited normal RVSF. Patients with reduced RVSF were more frequently male (76.1% vs. 70.5%, *P* = 0.027) and had higher rates of atrial fibrillation (18.6% vs. 6.8%, *P* < 0.001; *Table 1*). Acute coronary syndrome was less common in the reduced RVSF group (22.4% vs. 42.2%), while heart failure and cardiomyopathies were more prevalent (31.4% vs. 8.1%). Transthoracic echocardiograms were more often performed 1–30 days pre-angiography in the reduced RVSF group (48.6% vs. 33.8%, *P* < 0.001). Echocardiographic indices confirmed lower TAPSE (1.4 ± 0.4 vs. 2.0 ± 0.5 cm, *P* < 0.001) and elevated RA pressure (10.3 ± 6.0 vs. 7.6 ± 4.6 mmHg, *P* < 0.001). Invasive haemodynamics (*n* = 894) corroborated RV dysfunction with lower PAPi (66.5% vs. 83.3% with PAPi ≥ 2, *P* < 0.001) and reduced cardiac output (Qs Fick ≥ 4: 44.6% vs. 71.8%, *P* < 0.001).

**Table 1:** Baseline characteristics of the main study cohort

Variables	Reduced RVSF	Normal RVSF	*P*-value
Number coronary angiography studies, *n*	833	7220	—
Average videos per coronary angiography study, *n* (IQR)	7.0 (Q1: 5.0; Q3: 9.0)	9.0 (Q1: 7.0; Q3: 14.0)	—
Age (years), mean ± SD	66.5 ± 14.0	68.1 ± 12.0	0.11
Sex			
Male	634 (76.1%)	5091 (70.5%)	0.027
Female	198 (23.8%)	2127 (29.5%)	
Missing	1 (0.12%)	2 (0.02%)	
TTE			—
TAPSE (cm)	1.4 ± 0.4	2.0 ± 0.5	<0.001
RA pressure (mm Hg)	10.3 ± 6.0	7.6 ± 4.6	<0.001
Clinical haemodynamic index	215	679	—
PAPi ≥ 2	157 (66.5%)	585 (83.3%)	<0.001
PAPi < 2	58 (27%)	94 (14%)	
RA/PCWP ≥ 0.63	43 (22.4%)	90 (15.3%)	0.030
RA/PCWP < 0.63	149 (77.8%)	499 (84.7%)	
Qs Fick ≥ 4	100 (44.6%)	497 (71.8%)	<0.001
Qs Fick < 4	124 (55.4%)	205 (29.2%)	
Atrial fibrillation at the time of the procedure	155 (18.6%)	494 (6.8%)	<0.001
Coronary angiogram indication			—
ACS (% of angiograms)	187 (22.4%)	3054 (42.2%)	<0.001
STEMI, *n* (% of angiograms)	73 (8.8%)	884 (12.2%)	<0.001
NSTEMI/unstable angina, *n* (% of angiograms)	114 (13.7%)	2170 (30.1%)	<0.001
Non-ACS (% of angiograms)	646	4166	—
Ventricular arrhythmias	46 (7.1%)	182 (4.4%)	<0.001
Heart failure and cardiomyopathies	203 (31.4%)	332 (8.1%)	<0.001
Pre-valvular surgery	77 (11.9%)	602 (14.5%)	0.021
Ischaemic heart disease	94 (14.6%)	1312 (31.5%)	<0.001
Others	226 (35.1%)	1738 (41.7%)	<0.001
TTE timing relative to angiogram, *n* (% of angiograms)			
1–30 days pre, *n* (% of angiograms)	405 (48.6%)	2437 (33.8%)	<0.001
0–5 days post, *n* (% of angiograms)	428 (51.4%)	4783 (66.3%)	
Number of total coronary angiography videos, *n*	6752	75 368	—
Right coronary, *n* (% of angiograms)	2197 (32.5%)	24 501 (32.5%)	0.97
Left coronary, *n* (% of angiograms)	4555 (67.5%)	50 867 (67.5%)	
Brand			
Philips, *n* (% of angiograms)	774 (92.9%)	6550 (90.7%)	—
Siemens, *n* (% of angiograms)	39 (4.7%)	398 (5.5%)	—
Toshiba, *n* (% of angiograms)	20 (2.4%)	272 (3.8%)	—

Continuous data are expressed as mean ± SD, categorical data as *n* (%). Videos per patient is provided in quartiles format, indicating the median, 25th percentile (Q1), and 75th percentile (Q3).

NSTEMI, non-ST-segment elevation myocardial infarction; TTE, transthoracic echocardiogram; SD, standard deviation; STEMI, ST-segment elevation myocardial infarction; RA pressure, right atrial pressure; RA/PCWP, right atrial pressure to pulmonary capillary wedge pressure ratio; Qs Fick, systemic blood flow (calculated using the Fick principle); PAPi, pulmonary artery pulsatility index.

### Prediction of right ventricular systolic function on Montreal Heart Institute test data

**Figure 2:**
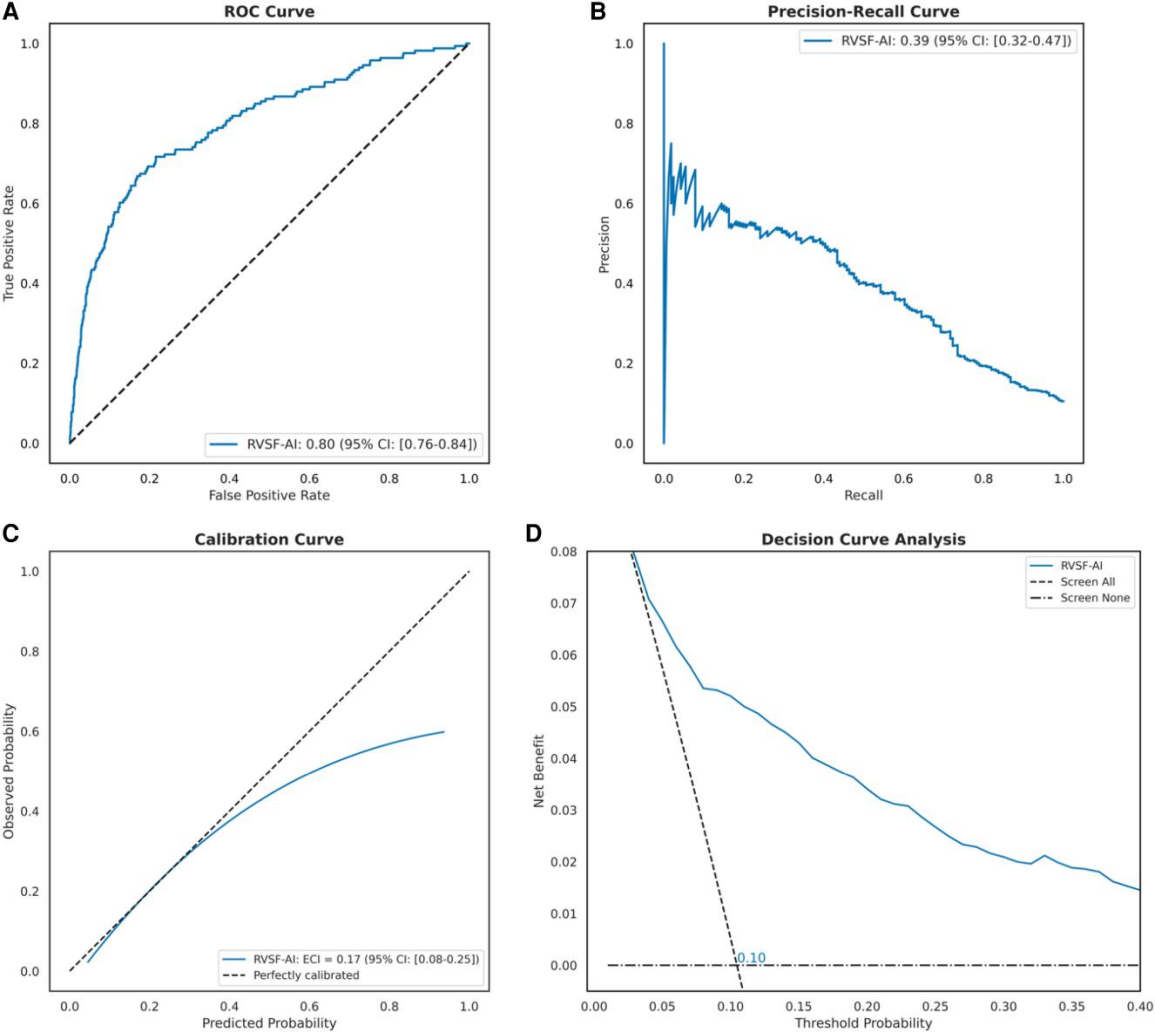
Performance assessment of DeepRV on the internal test set at the study level. (*A*) The ROC curve, plotting the true positive rate against the false positive rate for each model, with the area under the curve (AUROC) indicating discriminatory power. (*B*) The precision–recall curve, plotting precision against recall. (*C*) The calibration curve, showing the relationship between predicted and observed RVSF dysfunction risk; the slope and intercept are calculated using linear regression, and the curve is plotted using a univariate spline with smoothing factor of 1. The estimated calibration index (ECI, reported in the legend) is the root mean squared difference between the mean predicted probabilities and the spline-fitted calibration curve. (*D*) The decision curve analysis, plotting net benefit against threshold probability.

Upon evaluation of the holdout test set, the video-based DNN trained on both RCA and LCA videos demonstrated an AUROC of 0.80 (95% CI: 0.76–0.84) and an AUPRC of 0.39 (95% CI: 0.33–0.47; *Figure 2*; Supplementary material online, *Figure S3A* and *Table S5*). A calibration analysis showed an expected calibration index of 0.18 (95% CI: 0.11–0.26; Supplementary material online, *Figure S3B*). Further evaluation at Youden’s index (0.1) demonstrated a sensitivity of 70.5% (95% CI: 63.1–77.2), specificity of 78.5% (95% CI: 76.3–80.8), and a DOR of 8.73 (95% CI: 6.04–13.06; *Table 2*). Model performance was consistent across age and sex (see *Table 2*).

**Table 2:** Per-examination performance metrics by coronary dominance and vessel

	*n* (%)	Prevalence, *n* (%)	AUROC (95% CI)^a^	AUPRC (95% CI)^a^	DOR (95% CI)^a^	Sensitivity (95% CI)^a^	Specificity (95% CI)^a^	PPV (95% CI)^a^	NPV (95% CI)^a^
Study level (both LCA, RCA)	1586	166 (10.5%)	0.80 (0.76–0.84)	0.39 (0.33–0.47)	8.73 (6.04–13.06)	70.5 (63.1–77.2)	78.5 (76.3–80.8)	27.7 (23.5–31.6)	95.8 (94.5–96.9)
LCA only	1561	164 (10.5%)	0.77 (0.73–0.82)	0.35 (0.30–0.43)	6.82 (4.89–9.74)	72.0 (65.0–78.6)	72.7 (70.2–74.9)	23.6 (20.0–27.4)	95.7 (94.4–96.8)
RCA only	1363	137 (10.1%)	0.76 (0.71–0.81)	0.32 (0.26–0.40)	6.16 (4.20–9.01)	66.4 (58.6–74.3)	75.7 (73.3–78.2)	23.4 (18.9–27.7)	95.3 (93.9–96.6)
Right coronary dominance	1306 (95.5%)	156 (10.3%)	0.77 (0.73–0.80)	0.33 (0.29–0.38)	6.34 (4.84–8.27)	68.9 (63.7–74.4)	74.1 (72.4–75.8)	23.0 (20.2–25.9)	95.5 (94.6–96.5)
Left coronary dominance	62 (4.5%)	10 (14.3%)	0.79 (0.65–0.90)	0.41 (0.28–0.62)	9.56 (3.29–46.82)	77.8 (57.1–95.2)	73.2 (64.6–82.0)	35.0 (20.6–50.0)	94.7 (89.2–98.8)
Right coronary dominance and RCA	1318 (84.4%)	129 (9.8%)	0.76 (0.72–0.82)	0.32 (0.27–0.42)	6.23 (4.30–9.56)	66.7 (58.4–74.3)	75.7 (73.3–78.1)	22.9 (18.9–26.9)	95.4 (94.2–96.7)
Left coronary dominance and RCA	45 (15.6%)	8 (17.8%)	0.74 (0.51–0.92)	0.38 (0.24–0.73)	5.19 (1.05–6.27)	62.5 (25.0–100.0)	75.7 (61.8–88.2)	35.7 (7.7–62.5)	90.3 (79.4–100.0)
Right coronary dominance and LCA	1491 (95.5%)	154 (10.3%)	0.77 (0.73–0.81)	0.35 (0.29–0.43)	6.45 (4.56–9.35)	70.8 (63.2–78.5)	72.7 (70.3–75.0)	23.0 (19.2–26.6)	95.6 (94.3–96.8)
Left coronary dominance and LCA	70 (4.5%)	10 (14.3%)	0.83 (0.66–0.94)	0.47 (0.29–0.74)	22.76 (4.44–47.33)	90.0 (66.6–100.0)	71.7 (60.0–82.3)	34.6 (16.7–53.8)	97.7 (92.7–100.0)

Values in parentheses indicate 95% confidence intervals, reported using bootstrapping with 1000 iterations.

DOR, diagnostic odds ratio; AUROC, area under the receiver operating characteristic curve; RCA, right coronary artery; LCA, left coronary artery.

^a^The cut-off for determining sensitivity, specificity, PPV, and NPV for DeepRV was 0.1.

Model discrimination for reduced RVSF varied by coronary artery disease (CAD) burden and distribution (*Table 3*). Performance was highest in the absence of obstructive CAD (*n* = 580; AUROC 0.84, 95% CI 0.79–0.89), remained robust with isolated LCA disease (*n* = 272; AUROC 0.79, 95% CI 0.69–0.89) or multivessel disease (*n* = 422; AUROC 0.79, 95% CI 0.71–0.86), and was lower with isolated RCA disease (*n* = 312; AUROC 0.72, 95% CI 0.59–0.82). Discrimination was similar by atrial fibrillation status (no AF: *n* = 1447; AUROC 0.78, 95% CI 0.73–0.82; AF: *n* = 139; AUROC 0.79, 95% CI 0.70–0.86). Performance was robust with no or mild TR (*n* = 1384; AUROC 0.79, 95% CI 0.75–0.84) but decreased with moderate or severe TR (*n* = 202; AUROC 0.72, 95% CI 0.64–0.80).

**Table 3:** Performance of DeepRV to identify dysfunction of RVSF of the test dataset

	*n* (%)	Prevalence, *n* (%)	AUROC (95% CI)^a^	AUPRC (95% CI)^a^	DOR (95% CI)^a^	Sensitivity (95% CI)^a^	Specificity (95% CI)^a^	PPV (95% CI)^a^	NPV (95% CI)^a^
Age									
Age < 50	116 (7.3%)	13 (11.2%)	0.73 (0.55–0.88)	0.30 (0.16–0.59)	5.25 (1.44–21.84)	63.6 (33.3–90.0)	75.0 (67.5–83.5)	19.4 (6.4–33.4)	95.6 (90.9–99.0)
Age 50–64	448 (28.3%)	39 (8.7%)	0.84 (0.77–0.90)	0.48 (0.36–0.63)	13.86 (6.87–36.63)	78.7 (66.7–89.3)	78.9 (74.8–82.7)	28.2 (20.7–36.1)	97.2 (95.4–98.8)
Age 65–74	512 (32.3%)	54 (10.5%)	0.79 (0.73–0.86)	0.45 (0.34–0.58)	9.01 (5.00–17.62)	68.9 (56.7–80.0)	80.3 (76.7–83.8)	31.1 (23.3–38.7)	95.2 (92.9–97.2)
Age 75+	510 (32.2%)	60 (11.8%)	0.79 (0.71–0.85)	0.34 (0.24–0.47)	6.11 (3.25–13.28)	65.9 (52.5–78.8)	76.0 (71.5–80.0)	25.0 (17.1–32.8)	94.8 (91.8–97.3)
Sex									
Male	1094 (68.9%)	121 (11.1%)	0.80 (0.75–0.84)	0.43 (0.35–0.52)	9.02 (5.99–15.25)	72.7 (64.0–80.6)	77.2 (74.5–80.0)	28.4 (23.5–33.5)	95.8 (94.4–97.2)
Female	492 (31.0%)	45 (9.1%)	0.81 (0.73–0.87)	0.34 (0.25–0.50)	7.95 (4.16–15.97)	64.4 (50.0–79.1)	81.4 (77.7–84.7)	25.9 (17.3–34.7)	95.8 (93.6–97.8)
Strata of acute coronary syndromes classification									
STEMI	179 (11.3%)	9 (5.0%)	0.65 (0.52–0.79)	0.14 (0.07–0.40)	0.94 (0.48–6.64)	11.1 (0.0–37.5)	88.2 (82.6–92.8)	5.9 (0.0–20.0)	93.8 (89.1–97.6
STEMI LCA	81 (45.3%)	2 (2.5%)	0.86 (0.78–0.94)	0.12 (0.08–0.25)	2.26 (0.80–17.67)	16.7 (8.3–50.0)	91.9 (85.4–96.9)	7.1 (4.2–16.7)	96.7 (92.7–99.4)
STEMI RCA	77 (43.0%)	5 (6.5%)	0.55 (0.36–0.74)	0.09 (0.07–0.23)	0.69 (0.29–3.09)	8.3 (4.5–25.0)	88.4 (81.0–95.2)	5.6 (3.3–12.5)	92.1 (85.4–97.8)
NSTEMI/unstable angina	441 (27.8%)	23 (5.2%)	0.76 (0.64–0.86)	0.24 (0.14–0.44)	7.19 (3.02–19.05)	56.5 (33.3–75.8)	84.7 (81.0–87.9)	16.9 (8.3–25.3)	97.3 (95.4–98.8)
Non-ACS	966 (61.0%)								
Arrhythmias^c^	42 (4.4%)	5 (11.9%)	0.89 (0.75–0.98)	0.57 (0.31–0.93)	14.33 (4.53–36.43)	91.7 (75.0–95.0)	56.6 (39.7–71.3)	25.0 (7.9–44.7)	97.7 (96.9–98.2)
Heart failure and cardiomyopathies	119 (12.3%)	46 (38.7%)	0.70 (0.56–0.82)	0.77 (0.67–0.86)	10.86 (3.96–71.01)	92.6 (84.3–98.9)	46.6 (34.9–57.4)	52.4 (41.6–62.7)	90.8 (81.9–98.6)
Valvular and prosthetic heart disease	113 (11.7%)	15 (13.3%)	0.85 (0.73–0.95)	0.50 (0.34–0.78)	11.57 (3.83–76.74)	84.4 (67.6–97.2)	68.2 (59.3–77.9)	30.0 (17.4–43.6)	96.4 (92.1–99.3)
Ischaemic heart disease	266 (27.5%)	21 (7.9%)	0.83 (0.76–0.89)	0.28 (0.14–0.46)	4.96 (1.97–13.63)	52.3 (32.4–72.9)	81.9 (77.4–86.8)	20.5 (10.6–31.5)	95.0 (92.1–97.5)
Others	426 (44.1%)	47 (11.0%)	0.75 (0.66–0.82)	0.30 (0.22–0.43)	6.16 (3.18–12.21)	67.7 (54.4–79.8)	74.6 (70.1–79.1)	25.2 (18.4–33.0)	94.8 (92.1–97.0)
Strata of obstructive CAD pattern									
RCA obstructive	312 (19.7%)	27 (8.7%)	0.72 (0.59–0.82)	0.27 (0.17–0.47)	4.78 (2.01–11.40)	51.8 (32.7–70.0)	81.6 (76.9–85.8)	21.6 (12.2–32.6)	94.5 (91.6–97.3)
LCA obstructive	272 (17.2%)	23 (8.5%)	0.79 (0.69–0.89)	0.33 (0.21–0.53)	10.98 (4.57–30.32)	60.4 (40.4–78.9)	87.8 (83.6–91.4)	32.2 (19.3–44.6)	95.9 (93.4–98.1)
Both LCA and RCA obstructive	422 (26.6%)	48 (11.4%)	0.79 (0.71–0.86)	0.45 (0.33–0.60)	6.87 (3.71–13.91)	70.4 (56.7–83.0)	74.3 (70.1–78.5)	26.3 (18.4–33.7)	95.1 (92.4–97.4)
Neither LCA nor RCA obstructive	580 (36.6%)	68 (11.7%)	0.84 (0.79–0.89)	0.44 (0.36–0.57)	12.43 (6.89–24.70)	80.4 (70.7–89.3)	75.1 (71.1–79.0)	30.3 (24.2–37.3)	96.6 (94.8–98.1)
Atrial fibrillation at the time of the procedure									
Absent	1447 (91.2%)	131 (9.1%)	0.78 (0.73–0.82)	0.34 (0.28–0.42)	7.74 (5.35–11.43)	64.1 (55.6–72.2)	81.2 (79.1–83.4)	25.4 (20.6–30.2)	95.8 (94.7–96.9)
Present	139 (8.8%)	35 (25.2%)	0.79 (0.70–0.86)	0.59 (0.47–0.73)	13.09 (4.09–37.78)	94.3 (85.7–100.0)	44.2 (35.3–54.2)	36.3 (26.8–46.2)	95.8 (88.6–100.0)
Strata of tricuspid regurgitation severity									
TR ≥ 2 (moderate or severe)	1384 (87.26%)	99 (7.2%)	0.79 (0.75–0.84)	0.30 (0.23–0.39)	7.96 (5.17–12.87)	64.6 (55.1–74.0)	81.3 (79.1–83.3)	1384 (87.26%)	99 (7.2%)
TR < 2 (none or mild)	202 (12.74%)	67 (33.2%)	0.72 (0.64–0.80)	0.61 (0.52–0.71)	4.08 (2.18–8.60)	79.1 (69.2–88.2)	51.9 (43.7–59.4)	202 (12.74%)	67 (33.2%)
Clinical haemodynamic index^b^									
PAPi ≥ 2	143 (82.2%)	38 (26.6%)	0.84 (0.76–0.90)	0.64 (0.51–0.77)	9.45 (4.09–24.17)	73.7 (60.5–87.2)	77.1 (68.8–84.4)	53.8 (40.8–67.3)	89.0 (82.5–94.8)
PAPi < 2	31 (17.8%)	14 (45.2%)	0.88 (0.74–0.98)	0.84 (0.68–0.98)	42.25 (5.62–108.00)	92.9 (76.4–100.0)	76.5 (55.6–94.4)	76.5 (56.2–94.5)	92.9 (78.6–100.0)
RA/PCWP ≥ 0.63	24 (16.4%)	7 (29.2%)	0.82 (0.61–0.96)	0.63 (0.42–0.92)	6.00 (1.17–36.40)	71.4 (33.3–100.0)	70.6 (47.6–93.8)	50.0 (20.0–80.0)	85.7 (63.6–100.0)
RA/PCWP < 0.63	122 (83.6%)	36 (29.5%)	0.90 (0.84–0.95)	0.80 (0.69–0.90)	23.42 (9.54–104.89)	86.1 (74.4–96.7)	79.1 (70.4–87.1)	63.3 (49.0–77.5)	93.2 (86.9–98.6)
Qs Fick ≥ 4	112 (64.4%)	22 (19.6%)	0.84 (0.75–0.92)	0.52 (0.39–0.71)	15.75 (5.59–71.00)	81.8 (65.0–95.5)	77.8 (69.9–85.7)	47.4 (31.7–63.6)	94.6 (88.9–98.8)
Qs Fick < 4	62 (35.6%)	30 (48.4%)	0.86 (0.76–0.94)	0.84 (0.75–0.93)	9.86 (3.33–48.02)	76.7 (61.3–92.0)	75.0 (59.4–90.0)	74.2 (57.9–88.0)	77.4 (61.3–92.6)
TTE timing relative to angiography									
TTE before catheterization	589 (37.2%)	87 (14.7%)	0.81 (0.75–0.86)	0.47 (0.36–0.58)	9.51 (6.85–21.16)	70.1 (56.9–89.0)	80.2 (58.8–92.3)	40.1 (28.4–59.8)	93.4 (90.6–96.8)
TTE same day as catheterization	220 (14.2%)	13 (5.9%)	0.76 (0.58–0.89)	0.16 (0.06–0.32)	7.62 (3.88–42.24)	76.9 (50.0–100.0)	69.6 (45.4–90.9)	13.7 (6.7–33.3)	98.0 (95.9–100.0)
TTE after catheterization	777 (48.9%)	58 (7.5%)	0.74 (0.65–0.81)	0.25 (0.16–0.38)	5.88 (4.20–16.74)	67.2 (44.2–82.3)	74.1 (64.4–94.0)	17.3 (12.4–38.0)	96.6 (94.9–98.2)

Where data was only available for subgroups of the full cohort, the subgroup sample size is denoted by *N*.

AUROC, area under the curve for the receiver operating characteristic; AUPRC, area under the precision–recall curve; NPV, negative predictive value; PPV, positive predictive value; MHI, Montreal Heart Institute; RA/PCWP, right atrial pressure to pulmonary capillary wedge pressure ratio; Qs Fick, systemic blood flow (calculated using the Fick principle); PAPi, pulmonary artery pulsatility index; TR, tricuspid regurgitation.

^a^The cut-off for determining sensitivity, specificity, PPV, and NPV for DeepRV was 0.1.

^b^Haemodynamic data were only available for subgroups of the full cohort. The cardiogenic shock subgroup was extremely small (*n* = 4).

^c^For small subgroups or zero events, PPV/NPV were estimated with Laplace smoothing (0.5 added to each cell) to avoid division by zero and overconfident intervals.

Performance also differed by ACS subtype and culprit vessel (*Table 3*). Overall discrimination was lower in STEMI (*n* = 179; AUROC 0.65, 95% CI 0.52–0.79), driven by RCA-culprit STEMI (*n* = 77; AUROC 0.55, 95% CI 0.36–0.74) vs. LCA-culprit STEMI (*n* = 81; AUROC 0.86, 95% CI 0.78–0.94). A replacement analysis swapping diagnostic RCA videos with post-PCI RCA videos improved AUROC in STEMI + RCA culprit from 0.55 to 0.65, with minimal change in non-RCA culprit cases (0.86–0.90), implicating acute RCA occlusion as the key driver of reduced performance. NSTEMI/unstable angina showed intermediate discrimination (*n* = 441; AUROC 0.76, 95% CI 0.64–0.86). Performance decreased when TTE was performed post angiogram: pre-catheterization TTE yielded the highest discrimination (*n* = 589; AUROC 0.81, 95% CI 0.75–0.86), followed by same-day (*n* = 220; AUROC 0.76, 95% CI 0.58–0.89) and post-catheterization TTE (*n* = 777; AUROC 0.74, 95% CI 0.65–0.81).

For non-ACS indications, DeepRV achieved excellent discrimination in patients referred for angiography for investigation of ventricular arrhythmias (*n* = 42; AUROC 0.89, 95% CI 0.75–0.98) and pre-valvular surgery (*n* = 113; AUROC 0.85, 95% CI 0.73–0.95), good performance for ischaemic heart disease (*n* = 266; AUROC 0.83, 95% CI 0.76–0.89), and numerically lower performance for heart failure/cardiomyopathies (*n* = 119; AUROC 0.70, 95% CI 0.56–0.82) and other indications (*n* = 426; AUROC 0.75, 95% CI 0.66–0.82) (*Table 3*).

**Figure 3:**
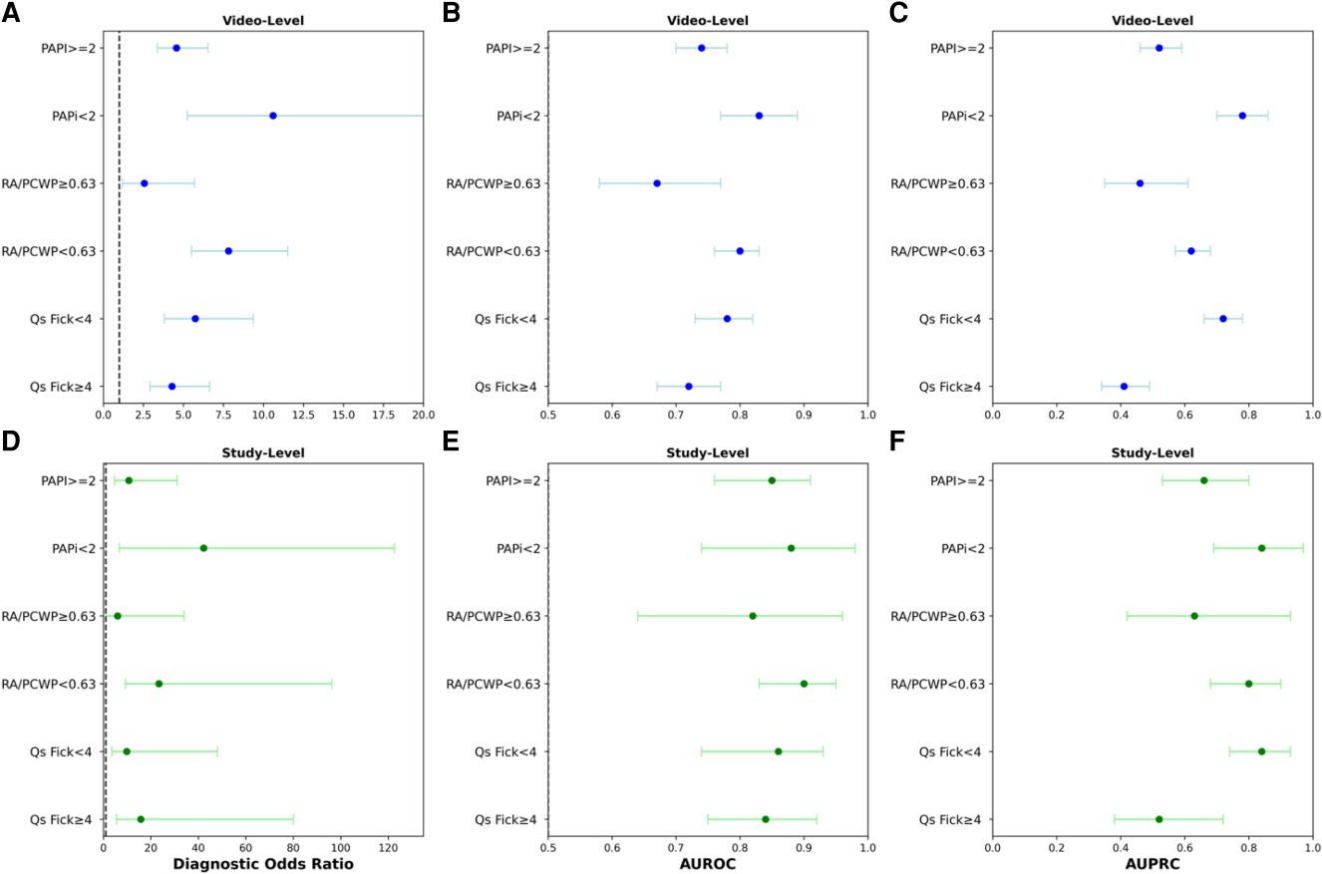
Discrimination performance metrics of DeepRV. Video-based deep neural network (DNN) to identify RVSF discrimination performance metrics on the videos and in subgroups of the MHI test set at the videos level (*A–C*) and at the exam level (*D–F*). (*A* and *D*) The diagnostic odds ratio, which is calculated as [sensitivity/(1 − sensitivity)]/[specificity/(1 − specificity)] at an optimal threshold of 10% for videos level and patient level. (*B* and *E*) The receiver operating characteristic area under the curve (AUROC). (*C* and *F*) The precision–recall curve area under the curve (AUPRC). Confidence intervals for all metrics were derived from 1000 bootstrap iterations.

DeepRV demonstrated robust discrimination for reduced RVSF across the haemodynamic spectrum. In a subset with paired CAG and RHC (*n* = 174), the model maintained excellent performance regardless of haemodynamic status: normal right atrial pressure (RA) and pulmonary capillary wedge pressure ratio (PCWP) < 0.63 (*n* = 122; AUROC 0.90, 95% CI 0.84–0.95), abnormal pulmonary artery pulsatility index (PAPi) < 2 (*n* = 31; AUROC 0.88, 95% CI 0.74–0.98), elevated RA/PCWP ≥ 0.63 (*n* = 24; AUROC 0.82, 95% CI 0.61–0.96), and reduced cardiac output ≤ 4 L/min (*n* = 62; AUROC 0.86, 95% CI 0.76–0.94; *Table 3* and *Figure 3*).

In patients with right coronary dominance, model performance was comparable whether the RCA (*n* = 1318; AUROC 0.76, 95% CI 0.72–0.82) or LCA (*n* = 1491; AUROC 0.77, 95% CI 0.73–0.81) was used as input (*P* = 0.68). However, in left-dominant circulation, LCA assessments yielded higher discriminatory performance (*n* = 70; AUROC 0.83, 95% CI 0.66–0.94) compared to RCA assessments (*n* = 45; AUROC 0.74, 95% CI 0.51–0.92; *Table 2*). At the angiographic view level, the best-performing projections were AP cranial (AUROC 0.74, 95% CI 0.71–0.77), LAO cranial (0.74, 0.69–0.78), and RAO straight (0.74, 0.68–0.80), followed by LAO caudal (0.73, 0.68–0.77). AP caudal showed the lowest discriminative performance (0.63, 0.56–0.70; Supplementary material online, *Table S6*).

### Cross-prediction analysis with CathEF

To test whether DeepRV captures RV-specific features beyond LV-RV coupling, we compared it to CathEF, our previous angiogram LVEF prediction for RVSF. Overall, CathEF achieved an AUROC of 0.807 (95% CI 0.768–0.842), confirming substantial LV-RV coupling. In patients with preserved LVEF (≥40%, *n* = 1341), DeepRV and CathEF achieved comparable performance (AUROC 0.706, 95% CI 0.629–0.772 vs. 0.738, 95% CI 0.676–0.800; *P* = 0.32). In patients with reduced LVEF (<40%, *n* = 241), DeepRV significantly outperformed CathEF (AUROC 0.759, 95% CI 0.692–0.815 vs. 0.661, 95% CI 0.589–0.732; *P* = 0.006). A combined model (DeepRV + CathEF) achieved the highest overall AUROC of 0.833 (95% CI 0.794–0.866).

### External validation of DeepRV

The external validation dataset comprised 2247 CAG studies from 2031 patients (mean age 65.5 ± 13 years, 66% male) at the University of California, San Francisco. Among these, 563 (27.7%) had reduced RVSF. Coronary angiography indications included arrhythmias (*n* = 58), heart failure/cardiomyopathies (*n* = 40), valvular/prosthetic disease (*n* = 24), and ischaemic heart disease (*n* = 13). DeepRV achieved an AUROC of 0.75 (95% CI: 0.72–0.77), sensitivity 71.3%, specificity 71.8%, PPV 50%, and NPV 86.6%. Calibration improved from ECI 0.23 to 0.037 after recalibration (see Supplementary material online, *Figures S5* and *S6*; *Table 4* and *Table S7*).

**Table 4:** Prospective study performance

	*n* (%)	Prevalence, *n* (%)	AUROC (95% CI)^a^	AUPRC (95% CI)^a^	DOR (95% CI)^a^	Sensitivity (95% CI)^a^	Specificity (95% CI)^a^	PPV (95% CI)^a^	NPV (95% CI)^a^
Diagnostic phase	82	10 (12.2%)	0.68 (0.49–0.86)	0.40 (0.19–0.68)	9.86 (1.82–57.70)	30.0 (0.0–60.0)	95.8 (90.5–100.0)	50.0 (0.0–100.0)	90.8 (83.8–96.2)
Post-PCI phase	100	13 (13.0%)	0.83 (0.71–0.93)	0.47 (0.30–0.77)	26.56 (4.41–122.32)	38.5 (11.1–66.7)	97.7 (94.1–100.0)	71.4 (27.2–100.0)	91.4 (84.6–96.7)

Values in parentheses indicate 95% confidence intervals, reported using bootstrapping with 1000 iterations.

DOR, diagnostic odds ratio; AUROC, area under the receiver operating characteristic curve; PCI, percutaneous coronary intervention.

^a^The cut-off for determining sensitivity, specificity, PPV, and NPV for DeepRV was 0.1.

### Prospective evaluation

**Figure 4:**
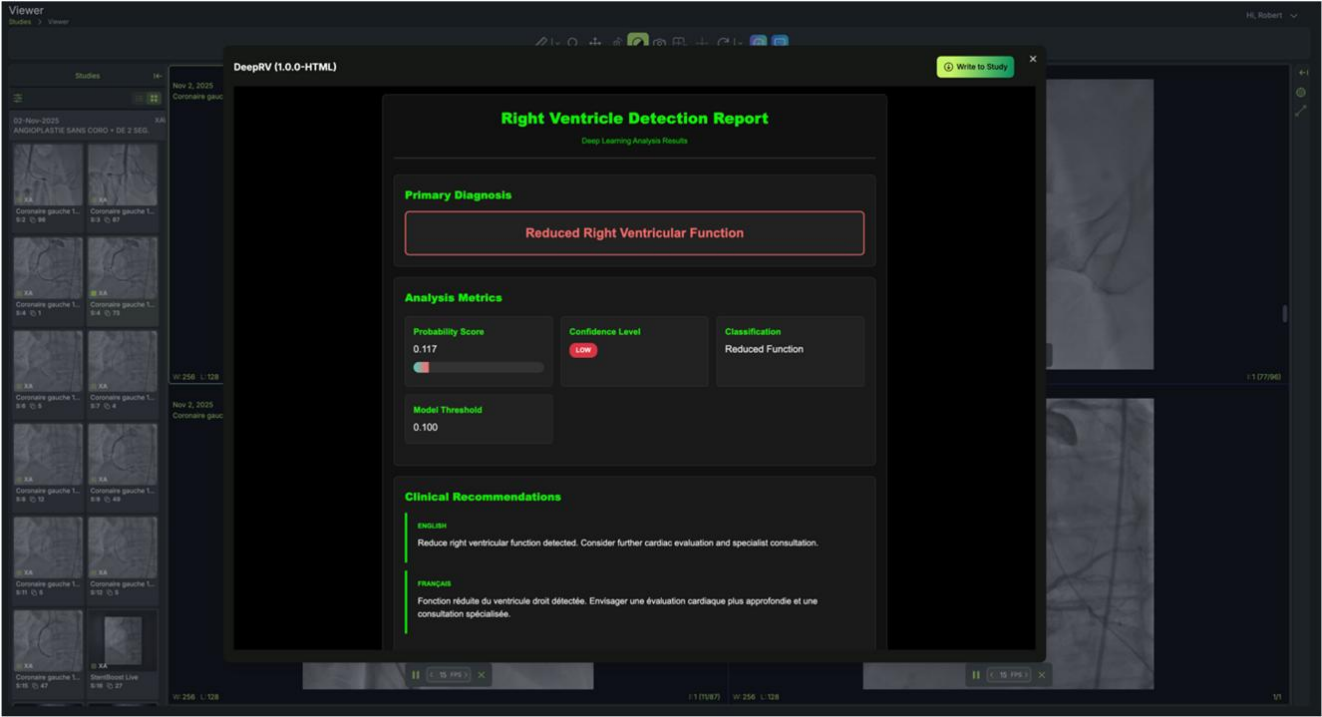
Clinical deployment of DeepRV in a patient with reduced right ventricular systolic function. DeepRV was executed directly from the catheterization laboratory PACS interface, generating an automated RV function estimate from the post-PCI angiogram within seconds. In this representative case, the model correctly identified reduced RV systolic function, aligning with the subsequent transthoracic echocardiogram. The workflow illustrates real-time, point-of-care inference and highlights operational readiness for integration into urgent coronary care pathways.

DeepRV was applied via PACS-AI (see Supplementary material online, *Figure S7*) successfully in 82 (100%) consecutive STEMI cases at our institution done between January and June 2025. Inference time took 5.1 s on median. The algorithm achieved an AUROC of 0.68 (95% CI: 0.49–0.86) when using angiogram images obtained during the initial diagnostic phase and an AUROC of 0.83 (95% CI: 0.71–0.93) when using the final angiograms, obtained in the post-PCI phase (*Figure 4*). Transthoracic echocardiograms were performed within 48 h in all cases. The NPV was similar (90.8% and 91.4%) respectively between the diagnostic and post-PCI phase, whereas the PPV was lower for the diagnostic phase than the post-PCI phase (50.0% vs. 71.4%).

### Human performance evaluation

In the reader study (*n* = 200 studies), AI alone achieved the highest accuracy (79.5%) and sensitivity (70.0%). Artificial intelligence assistance improved diagnostic accuracy for cardiologists (72.1–77.6%, +5.5 percentage points) and students (43.5–64.0%, +20.5 percentage points). Cardiologists with AI achieved the highest specificity (84.6%; *Figure 1*).

### Model interpretability

Grad-CAM analysis revealed that DeepRV primarily focused on coronary vessel motion during systole. Activation maps showed the highest attention to the dynamic displacement of both RCA and LCA during systolic phases, with minimal attention before contrast injection. This suggests the model learned to infer RV function from coronary motion patterns—the characteristic systolic displacement of vessels reflecting underlying cardiac mechanics (see Supplementary material online, *Video S1* and *Figure S8*).

## Discussion

DeepRV represents the first open-source automated assessment of RVSF from routine coronary angiograms using deep learning, transforming standard diagnostic procedures into dual-purpose tools that extract functional cardiac insights. We achieved moderate discrimination (AUROC 0.80 internal, 0.75 external) with excellent NPV (96%) but limited PPV (28%) and are releasing the model as open weights to accelerate research in this field. Importantly, AI assistance improved diagnostic accuracy across experience levels, with cardiologists improving from 72.1 to 77.6% and medical students demonstrating the largest gains (43.5–64.0%), while the human–AI combination achieved the highest specificity (84.6%). Our DNN performed well across most clinical scenarios, with notable exceptions. Performance was excellent for arrhythmias (AUROC 0.89) and valvular disease (AUROC 0.85) and remained robust even in patients with atrial fibrillation (AUROC 0.79), suggesting rhythm disturbances do not significantly impair the model’s ability to assess RVSF. Critically, DeepRV struggled with RCA-culprit STEMI (AUROC 0.55), while LCA-culprit STEMI showed excellent discrimination (AUROC 0.86). Model performance in STEMI patients was significantly influenced by the timing of both revascularization and echocardiography relative to angiography. In the prospective cohort of RCA and LCA STEMIs, DeepRV discrimination improved substantially when applied post-PCI (AUROC: 0.83) compared to pre-PCI imaging. This improvement reflects elimination of acute haemodynamic confounding from the culprit vessel occlusion, particularly in RCA STEMIs where acute ischaemia directly impairs RV contractility independent of baseline function. Importantly, model calibration remained robust despite substantial differences in disease prevalence across evaluation cohorts. Internal test set prevalence of RV dysfunction was 10.5%, compared to 27.6% in the external UCSF cohort, yet the model maintained clinically meaningful discrimination and the AUPRC scaled appropriately with prevalence (0.345 internally at 3.4× baseline vs. 0.579 externally at 2.1× baseline), confirming that the learned representations generalize beyond the training prevalence distribution.

The performance disparity between RCA-culprit STEMI and LCA-culprit STEMI reveals important mechanistic insights. Right coronary artery occlusion directly compromises RV perfusion through loss of acute marginal branches, causing immediate RV dysfunction that may not be fully captured by coronary flow dynamics alone during the diagnostic phase. In contrast, LCA-culprit STEMI preserves RV perfusion while potentially affecting RV function through altered septal mechanics and left ventricular interdependence, changes that may be more readily detected through coronary haemodynamics.^30,31^ As demonstrated in our prospective study, applying DeepRV to the post-PCI angiogram improves the classification performance to match the performance observed in our test set, which suggests RV recovery due to revascularization and stronger correlation with results found on TTE performed up to 48 h later.

While TTE remains the gold standard for RVSF assessment with established parameters (TAPSE, FAC, tissue Doppler), obtaining high-quality echocardiographic images in acute settings faces significant challenges, time constraints during urgent interventions, poor acoustic windows in critically ill patients, and mechanical ventilation interference.^32–35^ Moreover, assessing RV function relies on multiple parameters on TTE and is often challenging.^6,14,36,37^ Although our angiography-based model demonstrates slightly lower discrimination (AUROC 0.80) than recent echocardiography-based AI models (AUROC 0.84–0.87),^38^ DeepRV offers unique practical advantages due to its high NPV: it leverages routinely acquired coronary angiogram data for immediate rule out of RV dysfunction without additional imaging, minimizing delays. Moreover, in the post-PCI phase due to its high PPV of RVSF, DeepRV could guide targeted TTE evaluation during the hospitalization. DeepRV also demonstrated good discrimination based on our decision curve analysis and clinical utility, with calibration improving after recalibration in both datasets (MHI: ECI 0.18–0.17; UCSF: ECI 0.23–0.037), consistent with potential utility as a digital biomarker of RV function during angiography.

Coronary angiograms offer more than coronary disease evaluation; these data harbour subtle, ‘hidden to the human eye’ physiological biomarkers reflecting broader cardiac function, including LVEF and left ventricular end-diastolic pressure.^24,39^ Our team previously developed CathEF, a video-based DNN predicting LVEF from LCA angiograms^24^ (AUROC 0.911 for reduced LVEF, MAE 8.5% vs. TTE-LVEF). DeepRV extends this principle to the right ventricle, exploiting the fact that coronary motion patterns encode information about myocardial contractility beyond the left-sided chambers. The cross-prediction analysis in the present study substantiates this: while CathEF achieved comparable discrimination for RV dysfunction (AUROC 0.807), reflecting the strong biventricular interdependence expected in a catheterization population, DeepRV significantly outperformed CathEF in patients with reduced LVEF ≤ 40, where LV-derived signal alone is insufficient to resolve RV status. The combined CathEF-DeepRV model yielded the highest discrimination (AUROC 0.830), confirming that the two architectures encode complementary haemodynamic information and that simultaneous biventricular functional assessment from standard angiograms is feasible without additional imaging. Our explainability analyses provide mechanistic insight into how DeepRV captures these features: guided GradCAM^22^ showed minimal model attention during early pre-contrast frames, with activation intensifying as coronary arteries opacified, suggesting temporal awareness of contrast dynamics. This indicates the model leverages both structural (vessel geometry and displacement) and functional (contrast flow kinetics) cues in angiographic sequences to inform RV prediction.

Additionally, prior work has explored deep learning on ECG data for predicting RVSF dysfunction, which has achieved an AUROC with values ranging from 0.77 to 0.86 across different validation cohorts.^31^ Their model achieved AUROCs of 0.86 (95% CI 0.84–0.88) in the UK Biobank cohort, 0.81 (95% CI 0.78–0.84) in the Mount Sinai Hospital original cohort, and 0.77 (95% CI 0.65–0.88) in a prospective validation cohort.^31^ These studies have often focused on broader, general patient populations, with no description of performance in specific, acute clinical contexts such as ACS or STEMI patients. In these urgent scenarios, where rapid RVSF assessment is critical, but comprehensive imaging data might be initially lacking or delayed, the precise utility and reliability of ECG-based AI tools warrant further dedicated investigation. Future work could look at integrating ECG with DeepRV to improve model performance.^40–42^

Translating DeepRV into clinical practice can follow the pathway established by CathEF,^24^ which recently achieved an AUROC of 0.90 for detecting LVEF ≤ 40% in a cohort of 242 ACS patients undergoing primary PCI^43^ and was comparable to left ventriculography. This was achieved with the PACS-AI platform^43^ enabling real-time DICOM image transfer from acquisition systems directly to AI algorithms, providing immediate results within clinical workflows. DeepRV was deployed similarly through automated PACS connectivity. Angiographic images are routed to PACS in real time, where PACS-AI enables on-demand inference triggered by healthcare professionals during the procedure. This delivers RVSF assessments to the catheterization laboratory in near real time, demonstrating feasible integration of AI-based cardiac function evaluation into routine angiography. Optimal deployment strategy depends on clinical context and intended diagnostic threshold. To rule in RV dysfunction with high specificity, DeepRV should be applied during the diagnostic phase for elective cases and post-PCI for ACSs, the latter timing eliminates acute ischaemic confounding from culprit vessel occlusion. Conversely, for high-sensitivity rule-out applications, any angiographic phase is appropriate. For example, in anterior STEMI with suspected biventricular involvement, diagnostic-phase DeepRV could rapidly exclude significant RV dysfunction, to guide decisions regarding mechanical circulatory support escalation before intervention.^2^ A representative prospective case illustrates the clinical value: a 69-year-old woman presented with cardiac arrest and inferior STEMI on initial ECG which subsequently normalized. Despite an angiographically patent RCA and resolved ST-elevation, DeepRV flagged reduced RV function. We did not intervene on her RCA because she had triple-vessel disease and felt it was best to undergo heart team discussion. She deteriorated haemodynamically 6 h later requiring vasopressor support and TTE confirmed acute RV failure. After RCA stenting, the DeepRV score normalized applied via PACS-AI, demonstrating real-time detection of reversible RV dysfunction beyond standard angiographic assessment.

Although our DeepRV model was developed and validated on one of the largest angiographic datasets to date for automated RVSF assessment, several limitations should be acknowledged: (i) absence of race/ethnicity data, preventing assessment of algorithmic bias and fairness across demographic groups; (ii) lack of multimodality data (e.g. cardiac MRI, RHC) to validate RVSF assessments beyond echocardiography; (iii) temporal mismatch between angiography and TTE (up to 5 days), negatively affecting Deep RV performance in STEMI; (iv) small sample sizes for critical subgroups (cardiogenic shock *n* = 4, reduced PAPi *n* = 31); and (v) lack of core lab assessment of RVSF and potential inter-observer variability in TTE interpretation. These limitations set goals for future papers looking at improving the work on this type of biomarker. To facilitate this, we have provided the weights of the model for further validation and fine-tuning. Current evidence supports DeepRV for RVSF assessment in patients undergoing CAG, but its clinical utility varies by indication. The model demonstrates robust rule-out performance in stable populations, where high negative predictive value can obviate additional imaging. However, DeepRV should not replace TTE for comprehensive RV dysfunction screening in non-angiographic contexts. Critical gaps remain in acute settings—performance in isolated RV infarction and cardiogenic shock requires dedicated validation, as these haemodynamically extreme states may exceed the model’s training distribution.

## Conclusion

DeepRV enables automated RVSF assessment from routine coronary angiograms with moderate discrimination and high negative predictive value, supporting its use as a rule-out tool during catheterization. Artificial intelligence assistance improved diagnostic accuracy across experience levels. Prospective deployment confirmed real-time feasibility during primary PCI. The open-weight release enables external validation, fine-tuning, and integration with complementary modalities such as ECG-AI to advance automated biventricular assessment.

## Supplementary Material

ztag059_Supplementary_Data

## Data Availability

The datasets analysed during the current study are not publicly available due to patient privacy concerns but may be available from the corresponding author on reasonable request, as feasible and permitted by the Montreal Heart Institute institutional review board. DeepRV algorithm weights can be obtained at https://huggingface.co/heartwise/DeepCoro.

## Code availability

The underlying code for DeepRV inference for this study is available in a GitHub repository and can be accessible via the link https://github.com/HeartWise-AI/DeepRV.
