# Supplementary material for: Automated assessment of right ventricular systolic function from coronary angiograms with video-based artificial intelligence algorithms: development, validation, comparison against humans, and prospective deployment
Source: Eur Heart J Digit Health. 2026 Apr 15;7(4):ztag059. doi: 10.1093/ehjdh/ztag059 (PMC13174274; doi:10.1093/ehjdh/ztag059)
Supplement: ztag059_Supplementary_Data [file ztag059_supplementary_data.zip › Supplemental Materials_DeepRV_EHJDH.docx]

# SUPPLEMENTARY MATERIAL

**Methods**

**Study Data Sets and Study Population**

Raw angiographic data were obtained from Philips, Siemens, and Toshiba X-ray machines, in 512×512 pixels digital imaging and communications in medicine (DICOM) format. These files included angiographic views and video information from both right coronary and left CAG, with all patient identifiers removed. The average frame rate across all videos was 12.3 ± 3.8 frames per second (FPS), ranging from 7.5 to 15 fps. The closest TTE to the study date of the angiogram was used to assess the RVSF.

**Detailed Clinical, Echocardiographic, and Hemodynamic Variables**

**Clinical Data Collection** Electronic medical records were systematically reviewed to extract:

- Demographics: age, sex
- Primary indication for coronary angiography:
  - ACS: Determined through manual review of procedural notes and classified as STEMI or NSTEMI/unstable angina using keyword-based algorithms verified by human review
  - Non-ACS indications: arrhythmias, heart failure/cardiomyopathies, valvular/prosthetic heart disease, ischemic heart disease, other diagnostic indications
  - Note: No patients presented with cardiogenic shock as primary indication (n=4 had secondary diagnosis)
- Coronary dominance: Automatically determined using Swin3D neural network (AUROC 0.95) trained on publicly available dataset (19)

**Exclusion Criteria** No specific exclusions were applied for atrial fibrillation or pericardial disease; these were included as subgroup analyses.

**Echocardiographic Assessment** RVSF evaluation followed the 2025 American Society of Echocardiography guidelines (20):

- Primary measure: TAPSE from TTE reports (not core-lab adjudicated)
  - Normal: >17 mm
  - Mild dysfunction: 13-17 mm
  - Moderate dysfunction: 10-13 mm
  - Severe dysfunction: ≤10 mm
- Secondary measures when available: RV S' velocity, visual assessment, fractional area change (FAC), RV free wall strain
- Binary classification for model: Normal (>17mm) vs Reduced (≤17mm)
- Note: Updated classification per March 2025 ASE guidelines differs from prior cutoffs

**Hemodynamic Parameters (subset analysis)** For patients with right heart catheterization (n=224 internal, n=174 test set):

- Pulmonary Artery Pulsatility Index (PAPi): (PA systolic - PA diastolic)/mean RA pressure
  - Normal: ≥2.0
  - Abnormal: <2.0 (predictor of RV failure post-inferior MI)
- RA/PCWP ratio:
  - Normal: <0.63
  - Abnormal: ≥0.63 (indicates RV-predominant failure)
- Cardiac output (Fick method):
  - Normal: ≥4 L/min (consider indexing to BSA in future studies)
  - Reduced: <4 L/min

**Rationale for Hemodynamic Validation** Given limited sample size for some hemodynamic parameters (e.g., PAPi available in only 174 test patients), these served as exploratory validation rather than primary outcomes. The correlation between angiographic predictions and invasive hemodynamics provides mechanistic insight into model performance.

## Algorithm Development, training process:

In our study, we employed the X3D (1) model architecture, specifically the x3d_m variant, recognized for its efficiency in video-based learning tasks. We modified the original architecture by replacing the final classification layer with batch normalization followed by a linear layer outputting a single logit, suitable for binary classification using the binary cross-entropy loss.

Our preprocessing pipeline included downsampling videos to 256×256 pixels, normalization based on training set statistics, and temporal padding to ensure consistent input lengths. The model was initialized with pretrained weights from the Kinetics400 (2) dataset and fine-tuned on our coronary angiography (CAG) dataset.

We adopted a two-stage optimization strategy: initially using AdamW with a learning rate of 1e-2, and a batch size of 8; followed by fine-tuning with stochastic gradient descent (SGD)(3) using a learning rate of 1e-5 and batch size of 4. To optimize performance, we employed binary cross-entropy with logits loss (4), a learning-rate plateau scheduler, and early stopping to prevent overfitting. Hyperparameter tuning was performed using Bayesian optimization through the Weights & Biases platform. We ensured input video frames were multiples of 8 to accommodate X3D architectural constraints.

Each training was conducted using a batch size of 4 across 20 epochs, each video clip was sampled as a fixed sequence of 48 consecutive frames. This window length balances temporal coverage with computational efficiency. Hyperparameter tuning was performed systematically on the validation dataset to optimize the loss function using Weight and Biases (28). All model development and training were implemented using PyTorch (version 2.6). We also conducted comprehensive architectural exploration, evaluating multiple video model architectures before selecting X3D as the optimal model for RVSF prediction (Supplementary Table 6).

For comparative analysis, we also trained R(2+1)D (5), MViT (6), and Swin3D (7) architectures. The final model was selected based on the lowest validation loss on the development set (Supplementary Table 6).

**Model interpretation**

To interpret our DeepRV’s decision-making process, we employed two complementary explainability techniques: Gradient-weighted Class Activation Mapping (Grad-CAM) (8) and Guided Backpropagation (9).

Grad-CAM provides coarse localization maps by computing the gradient of the target class (right ventricular systolic function) with respect to the final convolutional layer’s feature maps. These gradients are globally average-pooled to produce important weights, which are used to generate a heatmap overlaid on the original video frames—where red indicates high relevance and blue indicates low relevance.

Guided Backpropagation (9) offers a finer visualization by modifying the backward ReLU operation to retain only positive gradients. These highlights input features that positively influence predictions. We applied Grad-CAM on the final convolutional layer and implemented custom backward passes for ReLU layers for Guided Backpropagation.

Combined, these techniques offer a comprehensive view of model interpretability: Grad-CAM highlights spatial attention, and Guided Backpropagation reveals detailed discriminative features.

**External validation**

We conducted external validation using an independent cohort to evaluate model transportability. We evaluated model performance using multiple complementary metrics. Calibration was evaluated using calibration plots comparing predicted probabilities against observed outcomes, quantified by the Expected Calibration Index (ECI).

Because no dedicated validation set was available in the external dataset, we applied 5-fold cross-validation for recalibration. The dataset was partitioned into five folds, with four folds used to fit the spline calibration and the fifth used for evaluation. This process was repeated so that each fold served once as the test set, enabling an unbiased estimate of post-calibration performance while avoiding information leakage.

**Statistical Analysis**

DeepRV’s classification performance was evaluated on the MHI test set and external validation dataset using standard classification metrics, including area under the receiver operating characteristic curve (AUROC), area under the precision-recall curve (AUPRC), Sensitivity, Specificity, positive prediction value (PPV), negative predictive value (NPV) and diagnostic odds ratio (DOR) metrics. The Diagnostic Odds Ratio (DOR) represents the ratio between the odds of disease in individuals who test positive and the odds of disease in those who test negative (32).

We calculated an overall prediction for each study by averaging the predictions from all its associated videos. We then found the best probability threshold using the Youden Index on the validation dataset, aiming to maximize both sensitivity and specificity. This threshold converted the raw probability scores into binary classifications, enabling the calculation of final sensitivity, specificity, PPV, and NPV.

Model calibration was assessed via calibration curves and quantified using the Estimated Calibration Index (ECI) (33), defined as the root mean squared difference between predicted probabilities and the spline-smoothed calibration curve. Calibration was then approved via spline-based post-hoc recalibration (34) (Supplemental Methods), and decision curve analysis was performed by plotting net benefit across varying threshold probabilities against default strategies (35).

In our study, we employed the SplineCalib algorithm from the ML-Insights (12) package to assess and improve the calibration of our model. This spline-based approach, introduced by Lucena et al., uses smooth cubic polynomials to fit model predictions to true probabilities, offering advantages over traditional methods like Platt scaling (13) and isotonic regression (14,15). We first evaluated the initial calibration of our model using calibration plots. Subsequently, we applied SplineCalib to recalibrate the model's probability outputs, which helped produce more reliable and interpretable probabilities. The calibrated probabilities were then used to determine an optimal decision threshold for our classification task. To identify the optimal threshold, we used both the SplineCalib results and Youden's index (16), a widely used method that maximizes the sum of sensitivity and specificity. Through this process, we identified the optimal threshold to be 0.1. The established threshold of 0.1 was then used for all further analyses and interpretations of the model's predictions on study-level.

Decision curve analysis incorporated both discrimination and calibration metrics. The Net Benefit (NB) was calculated at different decision thresholds. For the 'intervention for none' approach, NB is zero as both true positives (TP) and false positives (FP) are 0. The 'intervention for all' strategy assumes no adverse effects from over-screening, with NB calculated by replacing (TP/N) with prevalence and (FP/N) with (1 - prevalence) (17). This framework helps contextualize model predictions in practical decision-making scenarios.

Subgroup analyses were conducted to assess performance across clinical and imaging variables, including sex, age, coronary dominance, acquisition vendor, and coronary artery characteristics. We also examined performance in patients with varying hemodynamic profiles, including PAPi, RA/PCWP ratio, reduced cardiac output, primary clinical indication for coronary angiography (ACS, arrhythmias, heart failure and cardiomyopathies, valvular and prosthetic heart disease, ischemic heart disease, and other indications), as well as strata defined by coronary occlusion, heart failure status, and the presence of atrial fibrillation. We then compared the model's performance for each severity categories (mild, moderate, and severe) individually against its performance in patients with no RVSF dysfunction. The AUROC was computed for each group.

Continuous variables are presented as mean ± standard deviation or median (Quartile 1: Q1; Quartile 3: Q3), as appropriate. All statistical tests were two-sided, with a p-value ≤ 0.05 considered statistically significant. Confidence intervals (CIs) are reported using bootstrapping with 1000 iterations.

Data analysis and visualization were performed using Python (version 3.8), and R software (version 4.4.2, Vienna, Austria) with the following libraries: scikit-learn (version 1.3.2), lifelines (version 0.27.8), matplotlib (version 3.7.5), seaborn (version 0.13.2), opencv-python (4.5.1), scikit-learn (0.22.2), and pROC package (1.17) and dplyr (1.0.7) in R.

# Supplemental Methods References:

1. Feichtenhofer C. X3D: Expanding Architectures for Efficient Video Recognition [Internet]. arXiv; 2020 [cité 29 nov 2024]. Disponible sur: http://arxiv.org/abs/2004.04730

2. Kay W, Carreira J, Simonyan K, Zhang B, Hillier C, Vijayanarasimhan S, et al. The Kinetics Human Action Video Dataset [Internet]. arXiv; 2017 [cité 27 déc 2024]. Disponible sur: http://arxiv.org/abs/1705.06950

3. [PDF] Stochastic Estimation of the Maximum of a Regression Function | Semantic Scholar [Internet]. [cité 3 mars 2025]. Disponible sur: https://www.semanticscholar.org/paper/Stochastic-Estimation-of-the-Maximum-of-a-Function-Kiefer-Wolfowitz/f4e3738a90f9cc806a25c8739e0d8b892ee7d1ff

4. [2304.07288] Cross-Entropy Loss Functions: Theoretical Analysis and Applications [Internet]. [cité 3 mars 2025]. Disponible sur: https://arxiv.org/abs/2304.07288

5. Karpathy A, Toderici G, Shetty S, Leung T, Sukthankar R, Fei-Fei L. Large-Scale Video Classification with Convolutional Neural Networks. In: 2014 IEEE Conference on Computer Vision and Pattern Recognition [Internet]. 2014 [cité 8 févr 2025]. p. 1725‑32. Disponible sur: https://ieeexplore.ieee.org/document/6909619

6. Li Y, Wu CY, Fan H, Mangalam K, Xiong B, Malik J, et al. MViTv2: Improved Multiscale Vision Transformers for Classification and Detection [Internet]. arXiv; 2022 [cité 14 nov 2024]. Disponible sur: http://arxiv.org/abs/2112.01526

7. Yang YQ, Guo YX, Xiong JY, Liu Y, Pan H, Wang PS, et al. Swin3D: A Pretrained Transformer Backbone for 3D Indoor Scene Understanding [Internet]. arXiv; 2023 [cité 18 oct 2024]. Disponible sur: http://arxiv.org/abs/2304.06906

8. Selvaraju RR, Cogswell M, Das A, Vedantam R, Parikh D, Batra D. Grad-CAM: Visual Explanations from Deep Networks via Gradient-based Localization [Internet]. arXiv; 2019 [cité 14 nov 2024]. Disponible sur: http://arxiv.org/abs/1610.02391

9. Mostafa S, Mondal D, Beck MA, Bidinosti CP, Henry CJ, Stavness I. Leveraging Guided Backpropagation to Select Convolutional Neural Networks for Plant Classification. Front Artif Intell [Internet]. 11 mai 2022 [cité 3 mars 2025];5. Disponible sur: https://www.frontiersin.org/journals/artificial-intelligence/articles/10.3389/frai.2022.871162/full

10. Van Calster B, Vickers AJ. Calibration of Risk Prediction Models: Impact on Decision-Analytic Performance. Med Decis Making. 1 févr 2015;35(2):162‑9.

11. Huang Y, Li W, Macheret F, Gabriel RA, Ohno-Machado L. A tutorial on calibration measurements and calibration models for clinical prediction models. Journal of the American Medical Informatics Association. 1 avr 2020;27(4):621‑33.

12. Lucena B. Spline-Based Probability Calibration [Internet]. arXiv; 2018 [cité 26 janv 2025]. Disponible sur: http://arxiv.org/abs/1809.07751

13. ResearchGate [Internet]. [cité 6 mars 2025]. (PDF) Probabilistic Outputs for Support Vector Machines and Comparisons to Regularized Likelihood Methods. Disponible sur: https://www.researchgate.net/publication/2594015_Probabilistic_Outputs_for_Support_Vector_Machines_and_Comparisons_to_Regularized_Likelihood_Methods

14. Zadrozny B, Elkan C. Obtaining calibrated probability estimates from decision trees and naive Bayesian classifiers. In 2001 [cité 6 mars 2025]. Disponible sur: https://www.semanticscholar.org/paper/Obtaining-calibrated-probability-estimates-from-and-Zadrozny-Elkan/4f67a122ec3723f08ad5cbefecad119b432b3304

15. Zadrozny B, Elkan C. Transforming classifier scores into accurate multiclass probability estimates. In: Proceedings of the eighth ACM SIGKDD international conference on Knowledge discovery and data mining [Internet]. New York, NY, USA: Association for Computing Machinery; 2002 [cité 6 mars 2025]. p. 694‑9. (KDD ’02). Disponible sur: https://dl.acm.org/doi/10.1145/775047.775151

16. Schisterman EF, Faraggi D, Reiser B, Hu J. Youden Index and the optimal threshold for markers with mass at zero. Stat Med. 30 janv 2008;27(2):297‑315.

17. Vickers AJ, Elkin EB. Decision curve analysis: a novel method for evaluating prediction models. Med Decis Making. 2006;26(6):565‑74.

# Supplemental Figures:

**Supplemental Figure 1: Main Study Cohort (Montreal Heart Institute)**


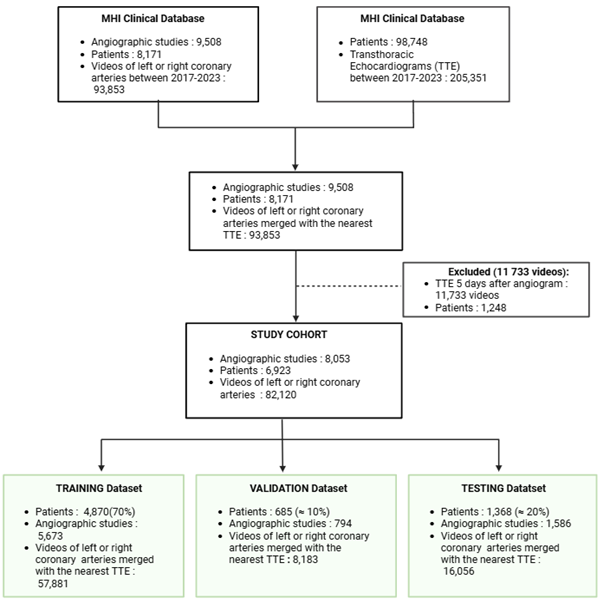
 Description of the inclusion of patients in the Main Study Cohort. Coronary angiograms performed at the Montreal Heart Institute from January 1^st^ 2016 and December 31^st^ 2023 that also had a TTE performed either up to one month prior to or within five days after the Coronary angiogram were obtained. Angiogram videos unsuitable for analysis were excluded, and the remaining cohort was randomly split by patient into training, development and test datasets for algorithm development and testing.

**
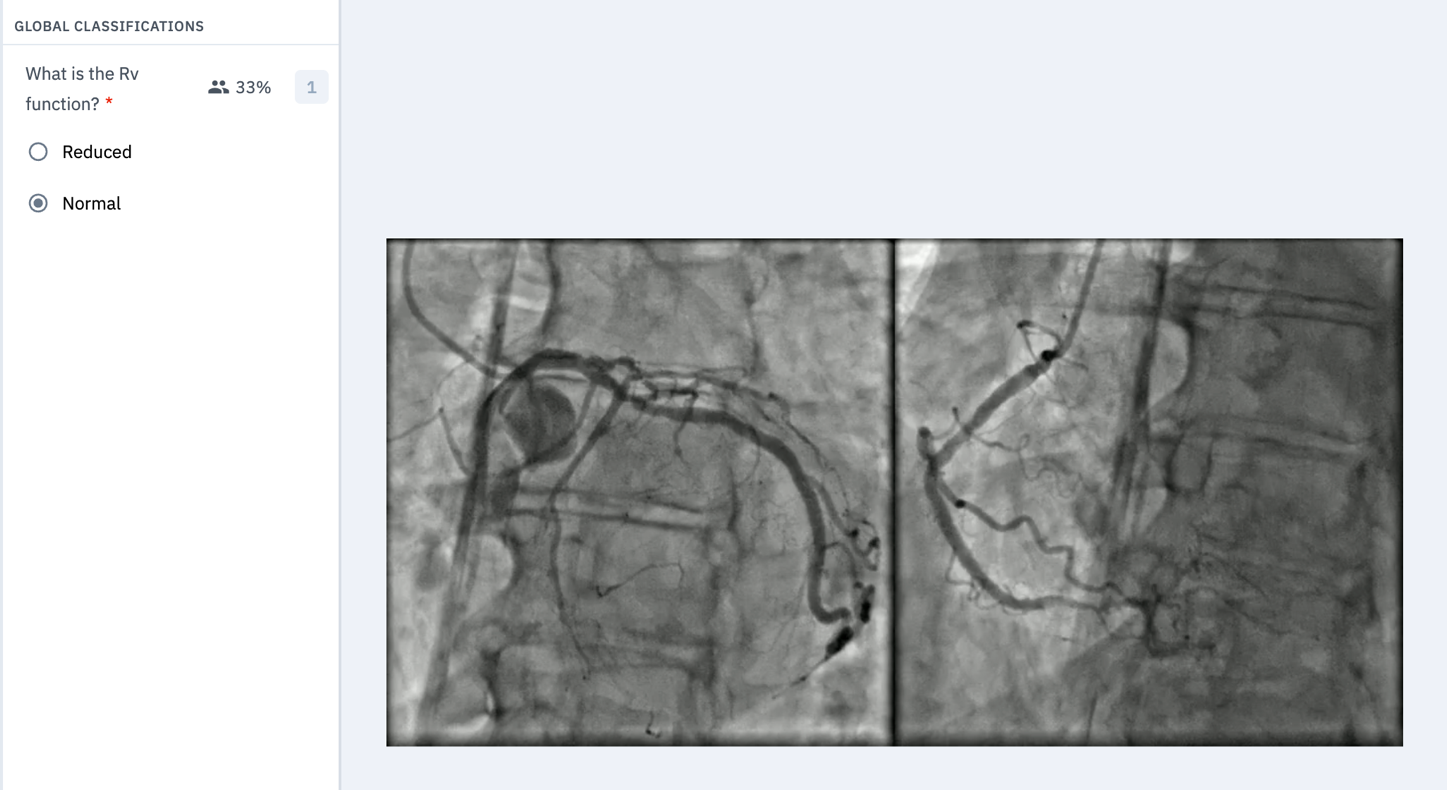
**

**Supplemental Figure 2. Annotation of DeepRV performance**

**Supplemental Figure 3a: Comparative performance assessment plots**


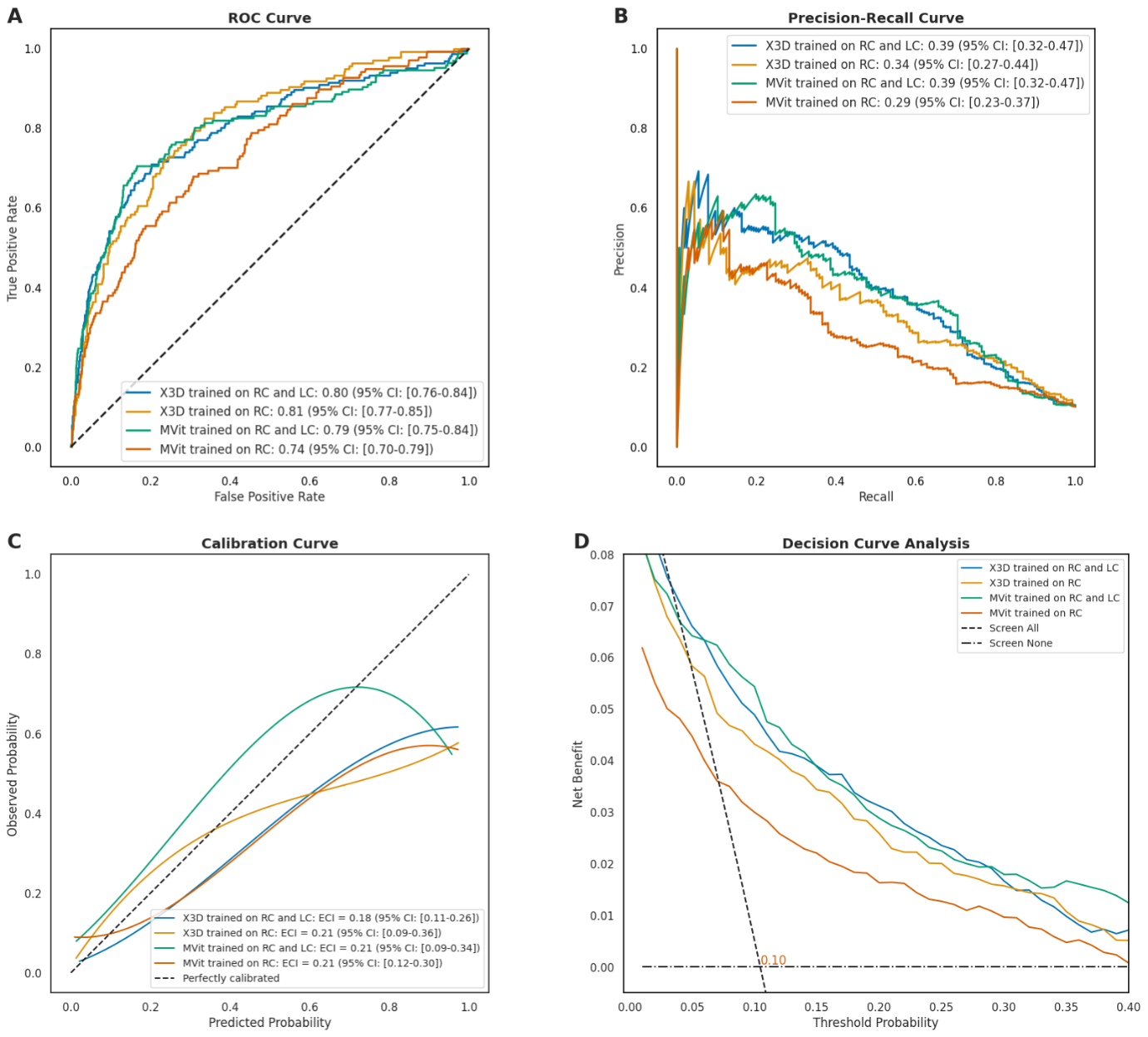
 MHI test set (1,568 Studies, 1,368 patients) performance assessment of the four models: (i) CAG-AI video-level based deep learning, (ii) CAG-AI patient-level, (iii)CAG-AI trained on CAG with angle value right coronary, (iv)and CAG-AI trained on CAG with angle value right coronary. (A) The receiver operating characteristic curve, plotting the true positive rate against the false positive rate for each model, with the area under the curve indicating discriminatory power and reported in the legend. (B) The precision–recall curve, plotting precision against recall, with the area under the curve reported in the legend. (C) The calibration curve, showing the relationship between predicted and observed RVSF dysfunction risk; the slope and intercept are calculated using linear regression, and the curve is plotted using a univariate spline with smoothing factor of 1. The estimated calibration index (ECI, reported in the legend) is the root mean squared difference between the mean predicted probabilities and the spline-fitted calibration curve. (D) The decision curve analysis, plotting net benefit against threshold probability.

**Supplemental Figure 3b: DeepRV performance after calibration**


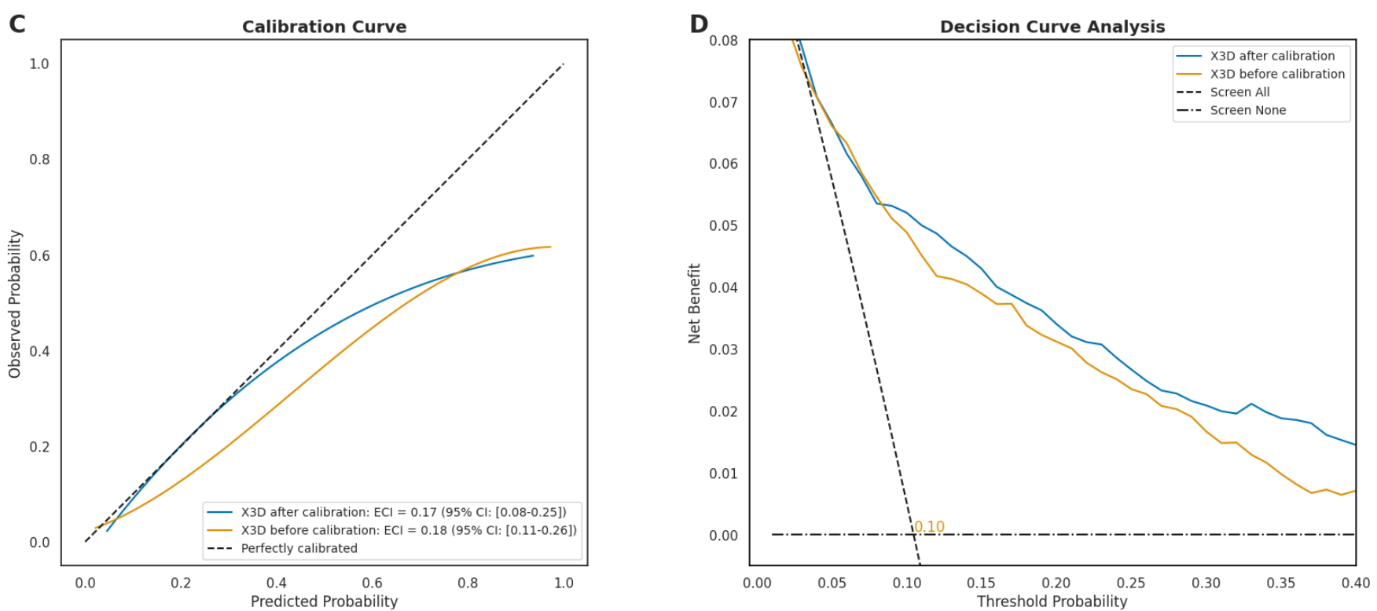
 MHI test set performance assessment of the model before calibration and after calibration. Panel C) The calibration curve, showing the relationship between predicted and observed RVSF dysfunction risk; the slope and intercept are calculated using linear regression, and the curve is plotted using a univariate spline with smoothing factor of 1. The estimated calibration index (ECI, reported in the legend) is the root mean squared difference between the mean predicted probabilities and the spline-fitted calibration curve. Panel D) The decision curve analysis, plotting net benefit against threshold probability.

**Supplemental Figure 4. Model performance in discriminating against different severities of RVSF dysfunction**:


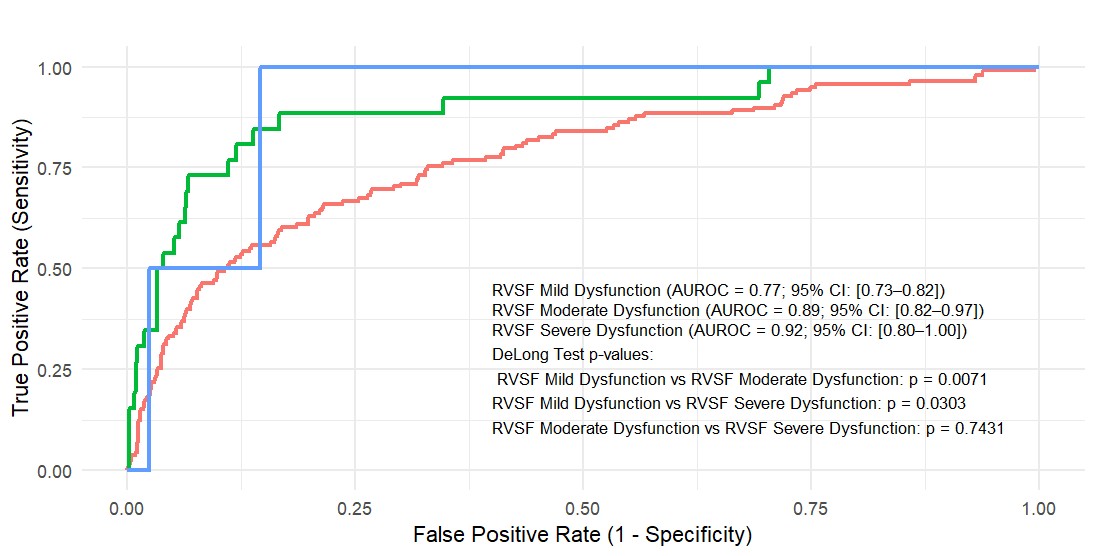


Receiver operating characteristic (ROC) curves stratified by the severity of RVSF dysfunction. Curves are shown for mild (red), moderate (green), and severe (blue) dysfunction. P-values for pairwise comparisons were calculated using DeLong’s test.

**Supplemental Figure 5: Performance assessment of DeepRV on the external validation dataset:**


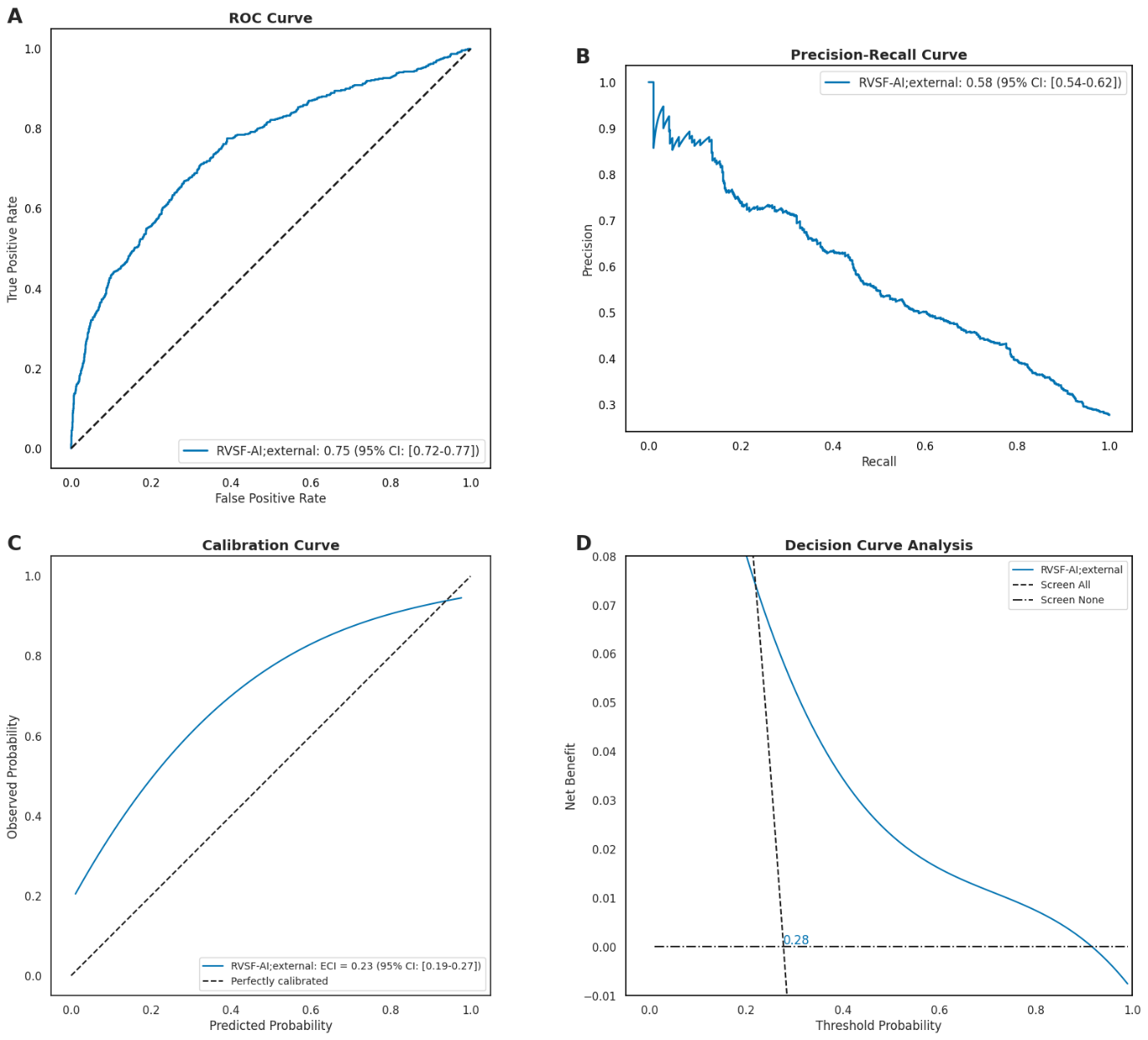
Panel A) shows the ROC Curve, plotting the True Positive Rate against the False Positive Rate for each model, with the area under the curve (AUC) indicating discriminatory power. Panel B) displays the Precision-Recall Curve, plotting precision against recall. Panel C) The calibration curve, showing the relationship between predicted and observed RVSF dysfunction risk; the slope and intercept are calculated using linear regression, and the curve is plotted using a univariate spline with smoothing factor of 1. The estimated calibration index (ECI, reported in the legend) is the root mean squared difference between the mean predicted probabilities and the spline-fitted calibration curve. Panel D) The decision curve analysis, plotting net benefit against threshold probability.

**Supplemental Figure 6: Performance assessment of DeepRV on the external validation data set recalibration:**


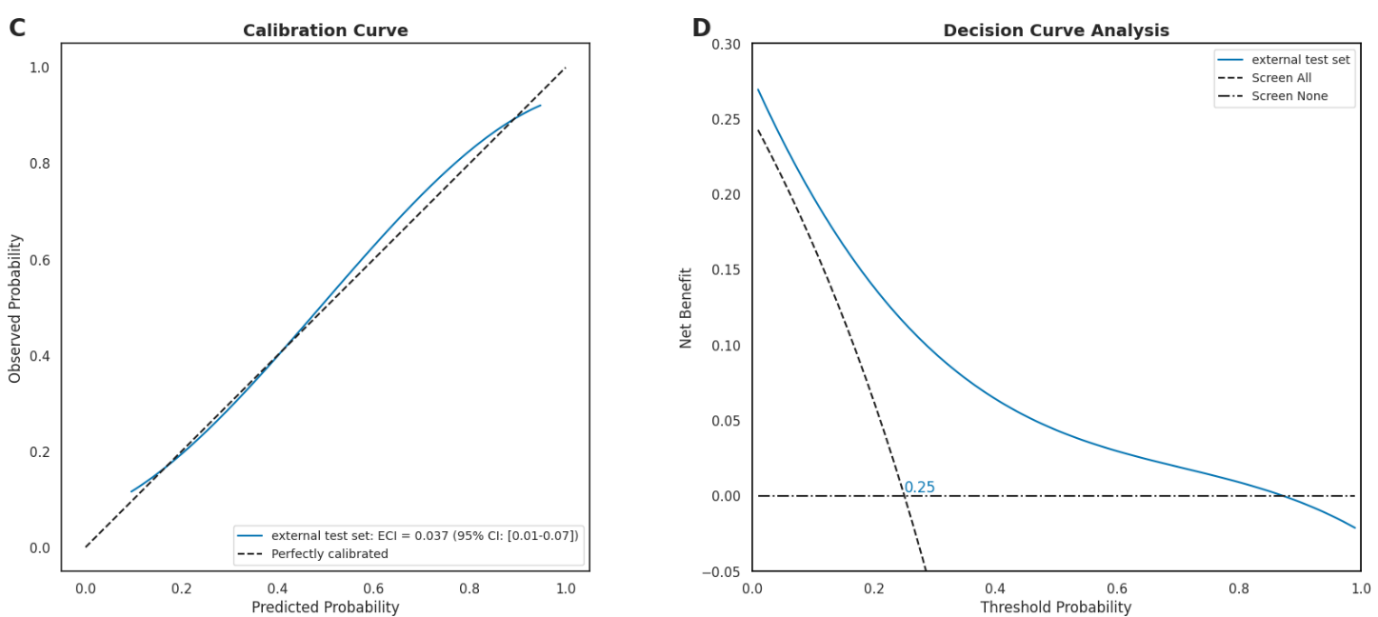
 (C) The calibration curve, showing the relationship between predicted and observed RVSF dysfunction; the slope and intercept are calculated using linear regression, and the curve is plotted using a univariate spline with smoothing factor of 1. The estimated calibration index (ECI, reported in the legend) is the root mean squared difference between the mean predicted probabilities and the spline-fitted calibration curve. (D) illustrates the decision curve analysis, plotting net benefit against threshold probability.

**Supplemental Figure 7: PACS-AI deployment of DeepRV:**


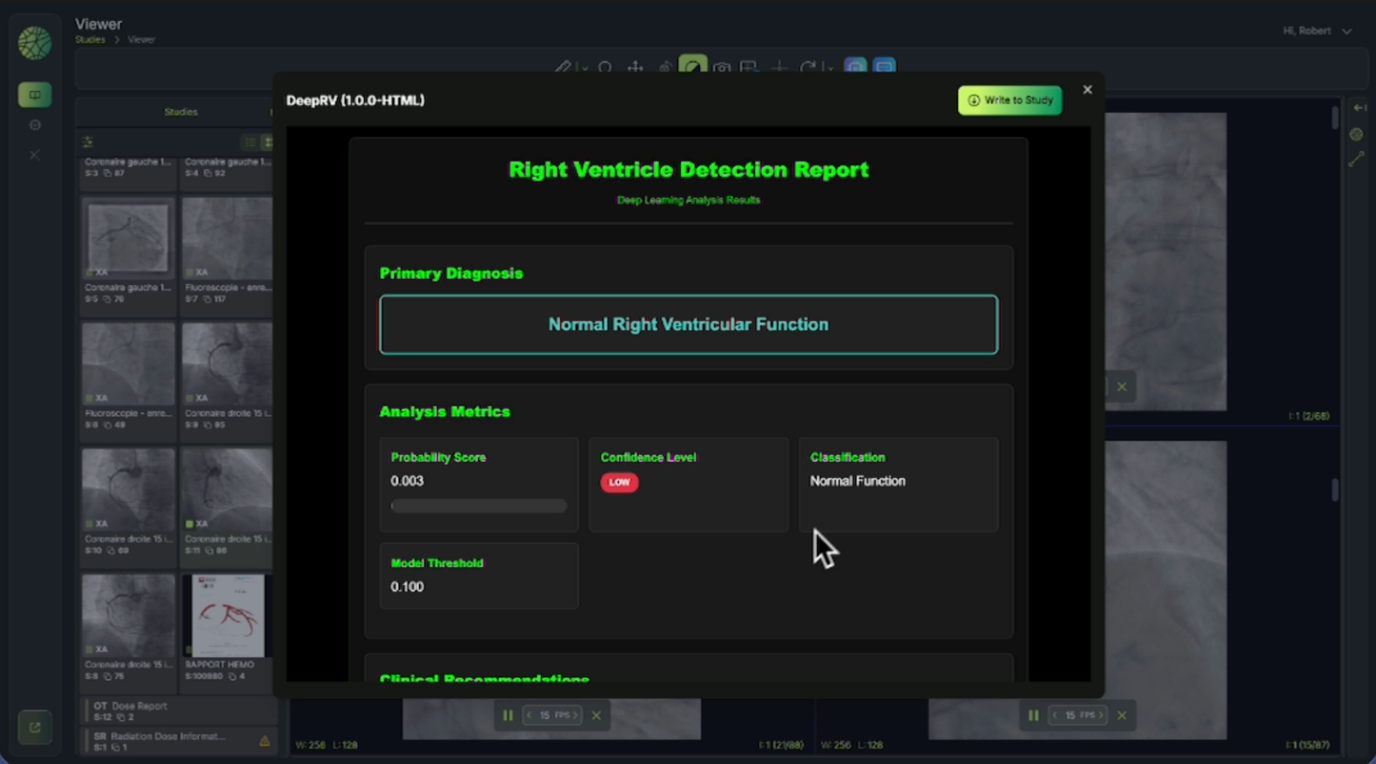


**Supplemental Figure 8: Comparison of Coronary Angiography Grad-CAM in Right and Left Dominant Coronary Circulation:**


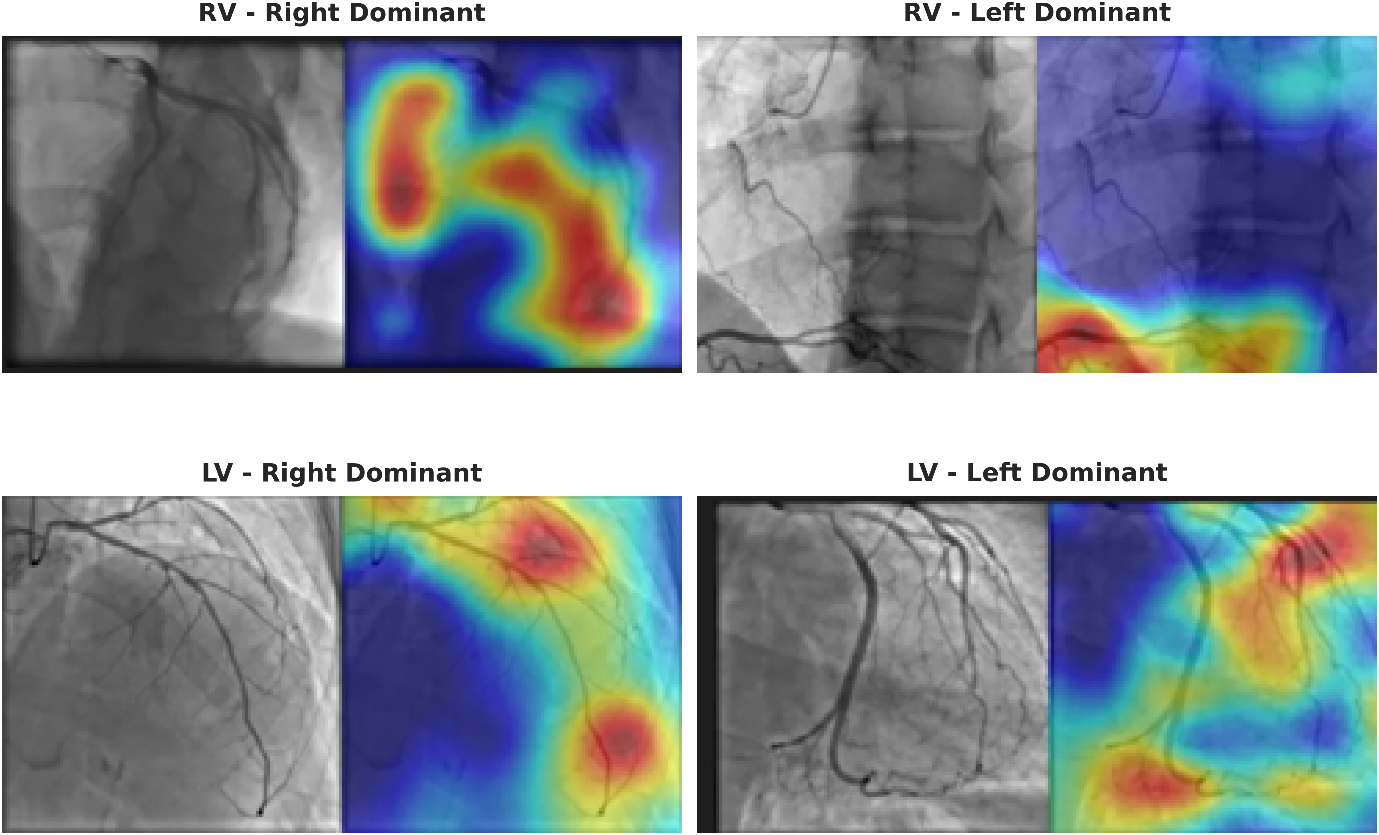


Representative examples of coronary angiograms (left panel of each pair) and corresponding guided GradCAM saliency maps (right panel of each pair) demonstrating the regions of pixel importance for the DeepRV model in predicting RVSF dysfunction. The GradCAM heatmaps indicate pixel contribution to the model's prediction, with warmer colors (red/yellow) signifying areas of higher importance and cooler colors (blue) indicating less importance.

**Supplemental Figure 9: GradCAM AI explainability technique applied to DeeppRV**

Supplemental video showing an angiogram video from a patient with right ventricular systolic function (RVSF) dysfunction (left), and the corresponding video comprised of guided GradCAM saliency maps (right). The guided GradCAM highlight pixels in each frame that contribute the most to DeepRV’s prediction of RVSF dysfunction in this video.

**Supplemental Tables**

**Table 1.** **Description of the MHI dataset overall and by split**

|  | Overall | Training | Validation | Test |
| --- | --- | --- | --- | --- |
| Number of total coronary angiography videos | **82,120** | **57,881** | **8,183** | **16,056** |
| videos per patient | 9.0 (Q1: 7.0; Q3: 15.0) | 9.0 (Q1: 7.0; Q3: 15.0) | 9.0 (Q1: 7.0; Q3: 15.0) | 9.0 (Q1: 7.0; Q3: 15.0) |
| Age [years] | 67.4 ± 12.0 | 67.3 ± 12.0 | 67.9 ± 12.0 | 67.4 ± 12.0 |
| Male | 59,142 (72.0%) | 42,136 (72.8%) | 5,761 (70.4%) | 11,245 (70.0%) |
| Female | 21,981 (26.8%) | 15,113 (26.1%) | 2,305 (28.2%) | 4,563 (28.4%) |
| Missing | 997 (1.2%) | 632 (1.1%) | 117 (1.4%) | 248 (1.5%) |
| MHI RVSF |  |  |  |  |
| None | 75,368 (91.8%) | 53,167 (91.9%) | 7,439 (90.9%) | 14,762 (91.9%) |
| Mild | 5,592 (6.8%) | 3,918 (6.8%) | 603 (7.4%) | 1,071 (6.7%) |
| Moderate | 1,025 (1.2%) | 691 (1.2%) | 136 (1.7%) | 198 (1.2%) |
| Severe | 1.35 (0.2%) | 105 (0.2%) | 5 (0.1%) | 25 (0.2%) |
| Number of total patients | **6,923** | **4,870** | **685** | **1,368** |
| Age [years] | 67.9 ± 12.2 | 67.9 ± 12.1 | 68.0 ± 12.3 | 67.6 ± 12.4 |
| Sex |  |  |  |  |
| Male | 4,820 (69.6%) | 3,417 (70.2%) | 469 (68.5%) | 934 (68.3%) |
| Female | 2,003 (28.9%) | 1,385 (28.4%) | 204 (29.8%) | 414 (30.3%) |
| Missing | 100 (1.4%) | 68 (1.4%) | 12 (1.8%) | 20 (1.5%) |
| MHI RVSF |  |  |  |  |
| None | 6,195 (89.5%) | 4,368 (89.7%) | 606 (88.5%) | 1,221 (89.3%) |
| Mild | 585 (8.5%) | 404 (8.3%) | 61 (8.9%) | 120 (8.8%) |
| Moderate | 128 (1.8%) | 85 (1.7%) | 18 (2.6%) | 25 (1.8%) |
| Severe | 15 (0.2%) | 13 (0.3%) | 0 (0.0%) | 2 (0.1%) |
| The number of videos per patient is provided in quartiles format, indicating the median,25th percentile (Q1), and 75th percentile (Q3).  Abbreviations: MHI: Montreal Heart Institute; RVSF: Right ventricle Systolic function. | | | | |

**Table 2. Definition of the angiographic projection angle classes**

| **Class** | **Definition** |
| --- | --- |
| RAO Cranial | -45**°** to -15**°** RAO; 15**°** to 45**°** Cranial |
| AP Cranial | -15**°** to 15**°** AP; 15**°** to 45**°** Cranial |
| LAO Cranial | 15**°** to 45**°** LAO; 15**°** to 45**°** Cranial |
| Straight RAO | -45**°** to -15**°** RAO; -15**°** to 15**°** AP |
| AP | -15**°** to 15**°** AP; -15**°** to 15**°** AP |
| RAO Caudal | -45**°** to -15**°** RAO; -45**°** to -15**°** Caudal |
| AP Caudal | -15**°** to 15**°** AP; -45**°** to -15**°** Caudal |
| LAO Caudal | 15**°** to 45**°** LAO; -45**°** to -15**°** Caudal |
| Straight LAO | 15**°** to 45**°** LAO; -15**°** to 15**°** AP |
| LAO Lateral | 70**°** to 110**°** LAO; -15**°** to 15**°** AP |
| RAO Lateral | -110**°** to -70**°** RAO; -15**°** to 15**°** AP |
| Other | Any angles not belonging to the previous definitions |
| **Abbreviation:** RAO = Right Anterior Oblique, LAO: Left Anterior Oblique, AP: Anteroposterior | |

**Table 3. Video-level performance of DeepRV to identify dysfunction of RVSF function of the test dataset**

|  | N (%) | | Prevalence  N (%) | AUROC  (95% CI) * | AUPRC  (95% CI) * | DOR  (95% CI) | Sensitivity  (95% CI) * | Specificity (95% CI) * | PPV  (95% CI) * | NPV (95% CI) * |
| --- | --- | --- | --- | --- | --- | --- | --- | --- | --- | --- |
| Video level † | | | | | | | | | | |
| Clinical hemodynamic index | | | | | | | | | | |
| PAPi≥2 | 910 (83.6%) | | 233 (25.6%) | 0.71 (0.69 - 0.73) | 0.22 (0.20 - 0.24) | 4.19 (3.71 - 4.73) | 55.5 (52.9 - 58.2) | 77.0 (76.3 - 77.7) | 16.8 (15.6 - 17.9) | 95.4 (95.1 - 95.8) |
| PAPi<2 | 179 (16.4%) | | 73 (40.8) | 0.83 (0.77 - 0.89) | 0.78 (0.71 - 0.86) | 10.60 (5.42 - 32.97) | 89.0 (81.2 - 95.5) | 56.6 (46.9 - 66.7) | 58.6 (49.5 - 67.5) | 88.2 (79.7 - 95.0) |
| RA/PCWP ≥0.63 | 157 (16.6%) | | 43 (27.4%) | 0.67 (0.58 - 0.77) | 0.46 (0.36 - 0.61) | 2.56 (1.23 - 6.30) | 69.8 (55.3 - 82.9) | 52.6 (43.4 - 61.4) | 35.7 (25.3 - 46.2) | 82.2 (73.0 - 90.1) |
| RA/PCWP<0.63 | 787 (83.3%) | | 216 (26.9%) | 0.78 (0.74 - 0.82) | 0.57 (0.51 - 0.63) | 7.18 (4.94 - 11.08) | 80.2 (74.4 - 85.3) | 63.9 (60.1 - 68.0) | 43.4 (38.4 - 48.6) | 90.3 (87.3 - 93.1) |
| Qs Fick ≥4 | 710 (63.8%) | | 136 (18.0%) | 0.71 (0.66 - 0.76) | 0.38 (0.32 - 0.47) | 4.24 (2.90 - 6.56) | 71.2 (63.3 - 78.7) | 63.1 (59.2 - 67.0) | 30.6 (25.3 - 35.9) | 90.6 (87.8 - 93.4) |
| Qs Fick <4 | 402 (36.2%) | | 178 (42.8%) | 0.76 (0.71 - 0.81) | 0.68 (0.62 - 0.75) | 5.27 (3.39 - 8.76) | 78.9 (72.7 - 85.1) | 58.5 (52.1 - 64.5) | 57.2 (50.9 - 63.6) | 79.8 (73.4 - 85.6) |
| All available videos from all projections | | 16,056 (100.0%) | 1,294 (8.0%) | 0.721 (0.707 - 0.737) | 0.242 (0.224 - 0.264) | 4.5 (4.0 - 5.0) | 57.4 (54.9 - 60.1) | 76.9 (76.2 - 77.6) | 31.8 (26.9 - 37.4) | 94.8 (93.7 - 96.1) |
| Age | | | | | | | | | | |
| Age<50 | | 1,238 (7.7%) | 76 (6.1%) | 0.88 (0.83 - 0.92) | 0.39 (0.30 - 0.50) | 20.93 (11.99 - 43.37) | 84.2 (75.4 - 92.6) | 79.7 (77.4 - 81.9) | 19.4 (6.4 - 33.4) | 95.6 (90.9 - 99.0) |
| Age 50-64 | | 4,852 (30.2%) | 316 (6.5%) | 0.77 (0.74 - 0.80) | 0.28 (0.24 - 0.33) | 5.80 (4.55 - 7.33) | 61.7 (56.2 - 67.1) | 78.3 (77.1 - 79.5) | 28.2 (20.7 - 36.1) | 97.2 (95.4 - 98.8) |
| Age 65-74 | | 5,114 (32.9%) | 428 (8.4%) | 0.70 (0.68 - 0.73) | 0.23 (0.20 - 0.27) | 4.10 (3.30 - 5.01) | 54.2 (49.5 - 58.9) | 77.6 (76.4 - 78.8) | 31.1 (23.3 - 38.7) | 95.2 (92.9 - 97.2) |
| Age 75+ | | 4,852 (30.2%) | 474 (9.8%) | 0.67 (0.64 - 0.70) | 0.23 (0.20 - 0.26) | 3.28 (2.73 - 4.01) | 53.4 (49.0 - 58.2) | 74.2 (72.8 - 75.5) | 25.0 (17.1 - 32.8) | 94.8 (91.8 - 97.3) |
| Sex | | | | | | | | | | |
| Male | | 11,428 (71.2%) | 1,294 (8.1%) | 0.73 (0.71 - 0.75) | 0.26 (0.23 - 0.28) | 4.63 (4.03 - 5.31) | 59.3 (56.4 - 62.7) | 76.1 (75.3 - 76.9) | 28.4 (23.5 - 33.5) | 95.8 (94.4 - 97.2) |
| Female | | 4,628 (28.8%) | 335 (7.2%) | 0.69 (0.66 - 0.72) | 0.20 (0.17 - 0.24) | 4.06 (3.21 - 5.05) | 51.9 (47.0 - 57.5) | 79.0 (77.7 - 80.2) | 25.9 (17.3 - 34.7) | 95.8 (93.6 - 97.8) |
| Atrial fibrillation at the time of the procedure | | | | | | | | | | |
| No | | 14,840 (92.4%) | 998 (6.7%) | 0.71 (0.69 - 0.73) | 0.20 (0.18 - 0.23) | 4.22 (3.70 - 4.85) | 53.4 (50.4 - 56.4) | 78.6 (77.9 - 79.3) | 15.3 (14.0 - 16.4) | 95.9 (95.5 - 96.3) |
| Yes | | 1,216 (7.7%) | 296 (24.3%) | 0.66 (0.62 - 0.69) | 0.42 (0.37 - 0.47) | 2.51 (1.94 - 3.43) | 70.9 (65.6 - 75.8) | 50.7 (47.2 - 54.1) | 31.6 (28.2 - 35.3) | 84.4 (81.4 - 87.5) |
| Strata of acute coronary syndromes classification | | | | | | | | | | |
| ACS | |  |  |  |  |  |  |  |  |  |
| STEMI | | 2,734 (17.0%) | 141 (5.2%) | 0.60 (0.56 - 0.64) | 0.07 (0.06 - 0.08) | 1.82 (1.25 - 2.58) | 29.8 (22.4 - 37.8) | 81.1 (79.6 - 82.6) | 5.9 (0.0 - 20.0) | 93.8 (89.1 - 97.6) |
| STEMI LCA | | 1,320 (48.3%) | 54 (4.1%) | 0.73 (0.68 - 0.79) | 0.08 (0.07 - 0.12) | 3.34 (1.90 - 5.83) | 42.7 (29.7 - 55.3) | 81.7 (79.7 - 83.9) | 9.2 (5.8 - 13.1) | 97.0 (96.0 - 98.0) |
| STEMI RCA | | 1,178 (48.1%) | 70 (5.9%) | 0.52 (0.45 - 0.59) | 0.06 (0.05 - 0.09) | 1.38 (0.75 - 2.33) | 26.1 (15.4 - 36.1) | 79.7 (77.4 - 82.2) | 7.6 (4.5 - 11.3) | 94.4 (92.9 - 95.8) |
| No angioplasty | | 236 (8.6%) | 17 (7.2%) | 0.49 (0.36 - 0.63) | 0.08 (0.06 - 0.14) | 0.49 (0.11 - 1.56) | 8.3 (2.2 - 21.9) | 84.3 (79.1 - 88.6) | 4.2 (1.1 - 11.3) | 91.8 (88.0 - 95.3) |
| NSTEMI / Unstable angina | | 4,899 (30.5%) | 192 (3.9%) | 0.72 (0.68 - 0.76) | 0.14 (0.10 - 0.18) | 4.66 (3.46 - 6.24) | 52.1 (45.0 - 58.7) | 81.1 (79.9 - 82.2) | 16.9 (9.2 - 25.9) | 97.3 (95.5 - 98.9) |
| Non_ACS | |  |  |  |  |  |  |  |  |  |
| Arrhythmias | | 313 (2.1%) | 32 (10.2%) | 0.72 (0.61 - 0.81) | 0.24 (0.17 - 0.38) | 3.98 (1.77 - 9.52) | 74.2 (58.6 - 88.3) | 58.0 (52.3 - 63.4) | 17.1 (11.5 - 23.3) | 95.1 (91.8 - 98.0) |
| Heart failure and cardiomyopathies | | 885 (5.5%) | 294 (33.2%) | 0.73 (0.69 - 0.76) | 0.59 (0.54 - 0.65) | 4.16 (3.10 - 5.65) | 75.4 (70.6 - 80.1) | 57.5 (53.4 - 61.3) | 46.9 (42.5 - 51.4) | 82.4 (78.8 - 86.2) |
| Valvular and prosthetic heart disease | | 777 (4.8%) | 97 (12.5%) | 0.72 (0.66 - 0.78) | 0.33 (0.26 - 0.42) | 4.54 (2.93 - 7.22) | 66.8 (57.2 - 75.8) | 69.2 (65.8 - 72.7) | 23.8 (18.9 - 29.0) | 93.6 (91.3 - 95.6) |
| Ischemic Heart Disease | | 2,502 (15.6%) | 187 (7.5%) | 0.65 (0.61 - 0.69) | 0.16 (0.13 - 0.20) | 2.87 (2.11 - 3.93) | 45.5 (38.1 - 52.6) | 77.5 (75.8 - 79.1) | 14.1 (11.4 - 16.8) | 94.6 (93.6 - 95.6) |
| Others | | 3,946 (24.6%) | 351 (8.9%) | 0.72 (0.69 - 0.74) | 0.23 (0.20 - 0.27) | 4.02 (3.18 - 5.00) | 58.4 (53.1 - 63.2) | 74.1 (72.7 - 75.6) | 18.1 (15.9 - 20.4) | 94.8 (94.0 - 95.6) |
| Strata of obstructive CAD pattern | |  |  |  |  |  |  |  |  |  |
| RCA obstructive | | 3,675 (22.9%) | 291 (7.9%) | 0.65 (0.61 - 0.68) | 0.16 (0.14 - 0.20) | 2.81 (2.15 - 3.58) | 43.7 (38.0 - 49.4) | 78.4 (77.0 - 79.8) | 14.8 (12.5 - 17.0) | 94.2 (93.2 - 95.0) |
| LCA obstructive | | 3,412 (21.3%) | 190 (5.6%) | 0.66 (0.62 - 0.71) | 0.13 (0.11 - 0.17) | 3.35 (2.52 - 4.53) | 43.2 (36.1 - 50.3) | 81.5 (80.2 - 82.8) | 12.2 (9.6 - 14.5) | 96.0 (95.3 - 96.8) |
| Both LCA and RCA obstructive | | 4,297 (26.8%) | 383 (8.9%) | 0.73 (0.70 - 0.76) | 0.27 (0.23 - 0.32) | 4.53 (3.66 - 5.69) | 61.1 (56.5 - 65.8) | 74.3 (72.9 - 75.6) | 18.9 (16.8 - 21.1) | 95.1 (94.3 - 95.8) |
| Neither LCA nor RCA obstructive | | 4,672 (29.1%) | 430 (9.2%) | 0.78 (0.75 - 0.81) | 0.33 (0.29 - 0.37) | 6.78 (5.38 - 8.55) | 69.7 (65.6 - 74.3) | 74.7 (73.3 - 76.1) | 21.8 (19.7 - 24.0) | 96.0 (95.4 - 96.7) |
| PCI Status | | | | | | | | | | |
| Right Coronary | | 2,270 (14.1%) | 127 (6.2%) | 0.61 (0.56 - 0.66) | 0.11 (0.08 - 0.16) | 2.20 (1.45 - 3.05) | 37.8 (29.9 - 45.3) | 78.4 (76.7 - 80.1) | 10.0 (7.6 - 12.7) | 95.2 (94.2 - 96.2) |
| Left Coronary | | 3,542 (22.1%) | 164 (4.6%) | 0.77 (0.73 - 0.81) | 0.18 (0.14 - 0.23) | 6.24 (4.58 - 8.76) | 57.9 (50.3 - 65.4) | 81.9 (80.7 - 83.1) | 13.5 (11.0 - 16.3) | 97.6 (96.9 - 98.1) |
| No PCI | | 10,244 (63.8%) | 995 (9.7%) | 0.72 (0.70 - 0.74) | 0.28 (0.25 - 0.30) | 4.43 (3.89 - 5.06) | 60.0 (57.2 - 62.9) | 74.7 (73.8 - 75.6) | 20.3 (18.9 - 21.8) | 94.6 (94.0 - 95.0) |
| Brand of angiograms | | | | | | | | | |  |
| Philips | | 14.476 (90.2%) | 1.160 (8.0%) | 0.71 (0.69 - 0.73) | 0.23 (0.21 - 0.25) | 4.23 (3.74 - 4.80) | 56.1 (53.3 - 59.1) | 76.8 (76.0 - 77.5) | 17.4 (16.2 - 18.7) | 95.3 (94.9 - 95.7) |
| Siemens | | 1,070 (6.7%) | 97 (9.1%) | 0.81 (0.76 - 0.85) | 0.43 (0.34 - 0.53) | 7.89 (5.20 - 12.93) | 66.0 (57.1 - 74.4) | 80.3 (77.9 - 82.9) | 25.0 (19.6 - 30.8) | 95.9 (94.5 - 97.3) |
| Toshiba | | 505 (3.2%) | 37 (7.3%) | 0.83 (0.75 - 0.89) | 0.31 (0.21 - 0.46) | 8.82 (4.36 - 24.15) | 75.7 (60.6 - 88.9) | 73.9 (70.0 - 77.8) | 18.7 (12.7 - 25.0) | 97.5 (95.6 - 99.1) |
| Anatomical structure | | | | | | | | | |  |
| Right coronary | | 5,145 (32.0%) | 398 (7.7%) | 0.72 (0.69 - 0.75) | 0.24 (0.20 - 0.28) | 4.74 (3.84 - 5.78) | 59.5 (54.8 - 64.3) | 76.3 (75.1 - 77.4) | 17.4 (15.4 - 19.3) | 95.7 (95.1 - 96.4) |
| Left coronary | | 10,911 (67.9%) | 896 (8.2%) | 0.72 (0.70 - 0.74) | 0.24 (0.22 - 0.27) | 4.39 (3.81 - 5.05) | 56.5 (53.2 - 59.7) | 77.2 (76.4 - 78.0) | 18.1 (16.7 - 19.5) | 95.2 (94.7 - 95.7) |
| Angiographic Projection | | | | | | | | | |  |
| RAO Cranial | | 477 (3.1%) | 15 (3.1%) | 0.68 (0.54 - 0.81) | 0.10 (0.05 - 0.25) | 2.19 (0.54 - 6.90) | 33.3 (10.0 - 60.0) | 81.4 (77.4 - 84.9) | 5.5 (1.1 - 10.3) | 97.4 (95.8 - 98.9) |
| AP Cranial | | 4,371 (27.2%) | 315 (7.2%) | 0.74 (0.71 - 0.77) | 0.26 (0.22 - 0.31) | 5.18 (4.07 - 6.60) | 61.3 (56.0 - 66.7) | 76.6 (75.2 - 77.9) | 16.9 (14.7 - 19.2) | 96.2 (95.5 - 96.9) |
| LAO Cranial | | 1,795 (11.2%) | 144 (8.0%) | 0.74 (0.69 - 0.78) | 0.28 (0.22 - 0.35) | 4.61 (3.27 - 6.60) | 56.2 (47.5 - 64.3) | 78.2 (76.0 - 79.9) | 18.4 (14.9 - 22.0) | 95.3 (94.2 - 96.4) |
| RAO Straight | | 855 (5.3%) | 82 (9.6%) | 0.74 (0.68 - 0.80) | 0.29 (0.22 - 0.37) | 5.79 (3.60 - 9.67) | 62.2 (50.7 - 72.2) | 77.9 (75.0 - 80.7) | 23.0 (17.4 - 28.5) | 95.1 (93.3 - 96.7) |
| AP | | 107 (0.7%) | 9 (8.4%) | 0.66 (0.47 - 0.84) | 0.16 (0.10 - 0.34) | 2.58 (0.51 - 12.41) | 55.6 (20.0 - 87.5) | 67.3 (57.3 - 76.8) | 13.5 (4.5 - 25.0) | 94.3 (88.3 - 98.6) |
| LAO Straight | | 3,220 (20.1%) | 299 (9.3%) | 0.72 (0.69 - 0.75) | 0.25 (0.21 - 0.29) | 4.02 (3.17 - 5.15) | 59.5 (54.5 - 64.8) | 73.2 (71.6 - 74.8) | 18.5 (16.1 - 20.9) | 94.6 (93.7 - 95.5) |
| RAO Caudal | | 1,682 (10.5%) | 148 (8.8%) | 0.71 (0.66 - 0.75) | 0.25 (0.20 - 0.32) | 3.87 (2.72 - 5.51) | 54.7 (46.2 - 63.6) | 76.2 (74.2 - 78.4) | 18.2 (14.9 - 21.8) | 94.6 (93.3 - 95.8) |
| AP Caudal | | 1,470 (9.2%) | 88 (6.0%) | 0.63 (0.56 - 0.70) | 0.16 (0.11 - 0.24) | 3.21 (2.03 - 5.03) | 48.9 (38.4 - 58.5) | 77.1 (74.7 - 79.3) | 11.9 (8.6 - 15.4) | 95.9 (94.7 - 97.0) |
| LAO Caudal | | 1,754 (10.9%) | 167 (9.5%) | 0.73 (0.68 - 0.77) | 0.25 (0.21 - 0.32) | 5.43 (3.92 - 7.69) | 52.7 (45.3 - 60.1) | 83.0 (81.2 - 84.9) | 24.6 (20.4 - 29.3) | 94.3 (93.2 - 95.5) |
| RAO Lateral | | 270 (1.7%) | 26 (9.6%) | 0.68 (0.54 - 0.80) | 0.28 (0.17 - 0.48) | 5.54 (2.37 - 18.00) | 65.4 (45.8 - 83.3) | 74.6 (68.5 - 79.9) | 21.5 (12.8 - 31.0) | 95.3 (92.4 - 98.0) |
| Abbreviations: AUROC: Area Under Curve for the Receiver Operating Characteristic; AUPRC: Area Under the Precision-Recall Curve; NPV: Negative Predictive Value; PPV: Positive Predictive Value; MHI: Montreal Heart Institute; RCA: Right Coronary Artery; LCA: Left Coronary Artery; PAPi: Pulmonary Artery Pulsatility Index; RA/PCWP: Right Atrial Pressure to Pulmonary Capillary Wedge Pressure Ratio; Qs Fick: Systemic Blood Flow (calculated using the Fick principle); PCI : Percutaneous Coronary Intervention; PAPi: pulmonary artery pulsatility index. Where data was only available for subgroups of the full cohort, the subgroup sample size is denoted by N.  *The cutoff for determining sensitivity, specificity, PPV and NPV for DeepRV was 0.09.  †We report DeepRV performance at the video-level, whereby every angiogram video is treated independently, with RVSF derived from the matched TTE. | | | | | | | | | | |

| LCA Only | 1561 | 164 (10.5%) | 0.77 (0.73-0.81) | 0.35 (0.28-0.43) | 7.4 (5.4-10.8) | 67.1 (59.6-73.9) | 78.5 (76.4-80.8) | 26.8 (22.8-31.2) | 95.3 (94.1-96.4) |
| --- | --- | --- | --- | --- | --- | --- | --- | --- | --- |
| AP Caudal | 787 (49.6%) | 65 (8.3%) | 0.76 (0.70-0.83) | 0.37 (0.26-0.48) | 7.6 (4.7-13.8) | 66.2 (55.2-77.3) | 79.6 (76.5-82.7) | 22.6 (17.1-28.7) | 96.3 (94.7-97.7) |
| RCA Only | 1363 | 137 (10.1%) | 0.76 (0.71-0.80) | 0.28 (0.22-0.36) | 7.4 (5.2-11.3) | 65.7 (57.5-73.9) | 79.5 (77.3-81.7) | 26.4 (22.0-31.2) | 95.4 (94.1-96.6) |
| AP Cranial | 1441 (90.9%) | 138 (9.6%) | 0.75 (0.70-0.79) | 0.31 (0.24-0.40) | 6.7 (4.6-9.9) | 63.8 (55.6-71.8) | 79.1 (77.0-81.3) | 24.4 (19.9-29.2) | 95.4 (94.1-96.6) |
| LAO Lateral | 181 (11.4%) | 15 (8.3%) | 0.74 (0.61-0.86) | 0.20 (0.09-0.41) | 6.0 (2.0-23.2) | 60.0 (33.3-85.7) | 80.1 (73.8-86.0) | 21.4 (9.3-33.4) | 95.7 (92.2-98.6) |
| LAO Straight | 1371 (86.4%) | 141 (10.3%) | 0.74 (0.69-0.78) | 0.29 (0.23-0.37) | 4.9 (3.4-7.1) | 65.2 (56.9-73.3) | 72.1 (69.8-74.7) | 21.1 (17.5-25.1) | 94.8 (93.2-96.1) |
| RAO Caudal | 1024 (64.6%) | 108 (10.5%) | 0.73 (0.68-0.79) | 0.30 (0.23-0.40) | 5.7 (3.8-9.0) | 52.8 (43.6-62.8) | 83.6 (81.2-86.0) | 27.5 (21.9-34.2) | 93.8 (92.1-95.4) |
| RAO Straight | 673 (42.4%) | 60 (8.9%) | 0.72 (0.65-0.78) | 0.22 (0.14-0.33) | 4.7 (2.7-8.8) | 68.3 (56.5-80.0) | 68.4 (64.7-71.9) | 17.4 (12.7-22.1) | 95.7 (93.5-97.5) |
| LAO Cranial | 1020 (64.3%) | 99 (9.7%) | 0.71 (0.65-0.77) | 0.28 (0.20-0.37) | 5.4 (3.5-8.5) | 59.6 (49.5-68.9) | 78.6 (76.0-81.1) | 23.0 (18.1-28.5) | 94.8 (93.1-96.3) |
| Other | 433 (27.3%) | 37 (8.5%) | 0.69 (0.59-0.78) | 0.20 (0.12-0.33) | 5.8 (2.5-28.3) | 86.5 (75.0-97.1) | 47.5 (42.7-52.6) | 13.3 (9.0-17.7) | 97.4 (95.0-99.5) |
| RAO Cranial | 239 (15.1%) | 11 (4.6%) | 0.68 (0.50-0.83) | 0.11 (0.04-0.29) | 4.9 (1.3-24.4) | 63.6 (33.3-91.7) | 73.7 (67.4-79.0) | 10.4 (4.0-18.0) | 97.7 (95.3-99.5) |
| LAO Caudal | 1065 (67.2%) | 109 (10.2%) | 0.68 (0.62-0.73) | 0.23 (0.18-0.33) | 3.7 (2.5-5.6) | 56.9 (48.0-65.7) | 73.6 (70.8-76.4) | 19.7 (15.4-24.3) | 93.7 (92.0-95.4) |
| AP | 85 (5.4%) | 8 (9.4%) | 0.50 (0.30-0.70) | 0.10 (0.05-0.23) | Inf | 100.0 (100.0-100.0) | 18.2 (9.6-27.4) | 11.3 (4.3-19.1) | 100.0 (100.0-100.0) |

**Table 4. Performance of DeepRV to identify dysfunction of RVSF function of the external validation dataset**

|  | **AUROC  (95% CI)** | **AUPRC**  **(95% CI)** | **Sensitivity  (95% CI)** | **Specificity (95% CI)** | **PPV  (95% CI)** | **NPV (95% CI)** |
| --- | --- | --- | --- | --- | --- | --- |
| **Internal validation** | 0.80  (0.76, 0.84) | 0.39  (0.33- 0.47) | 60.2  (52.4 - 67.5) | 84.9  (83.2 - 86.7) | 31.8  (26.9 - 37.4) | 94.8  (93.7 - 96.1) |
| **External validation** | 0.75  (0.72, 0.77) | 0.58  (0.54-0.62) | 71.3  (67.31-75.4) | 71.8  (69.9-74.24) | 50  (46.2-52.9) | 86.6  (84.6-99.3) |
| **Abbreviations**: AUROC: Area Under Curve for the Receiver Operating Characteristic; AUPRC: Area Under the Precision-Recall Curve; NPV: Negative Predictive Value; PPV: Positive Predictive Value; | | | | | | |

**Table 5. Hyperparameters searched for** **DeepRV**

| **Parameters** | **Values** |
| --- | --- |
| **Number of frames** | 36; 48; 60; 72 |
| **Sampling of frames** | Every frame; Every other Frame |
| **Image Size** | 256x256 px |
| **Learning Rate** | 10e-2; 10e-3; 7e-4 |
| **Scheduler type**  **Factor**  **Patience**  **Threshold**  **Learning step period** | Step  0.3  9  0.01  15 |
| **Image Augmentation** | - None - Rotation - Horizontal Flipping - Skewing - Translation - Brightness and contrast variations |
| **Architecture** | - X3D |
| **Batch size** | 4, 8, 10 |
| **Optimizer** | AdamW, SGD |

**Table 6. Development set performance of alternative training schemes and**

**hyperparameters**

| **Model Architecture** | **Hyperparameters tuned** | **Best development set AUROC at the video-level** |
| --- | --- | --- |
| **X3D** (Model presented in the Manuscript) | Videos of the left and right coronary artery (256). - Learning rate (1e-4;1e-3) - SGD optimizer - Batch size 4 - No video augmentation  - Max epochs 20 - Frames: [48,64,72,80] - Loss: bce_logit_loss | **0.762** |
| **X3D (x3d_m)** using right coronary artery | Videos of the right coronary artery (256). - Learning rate (1e-4;1e-3) - SGD optimizer - Batch size 4 - No video augmentation - Max epochs 20 - Frames: [48,64,72,80] - Loss: bce_logit_loss | **0.760** |
| **X3D (x3d_m) with focal loss** | Videos of the left and right coronary artery (256). - Learning rate (1e-4;1e-3) - SGD optimizer - Batch size 4 - No video augmentation - Max epochs 20 - Frames: [48,64,72,80] - Loss: focal_loss | **0.735** |
| **MViT (mvit_v2_s)** | Videos of the left and right coronary artery (224x224). - Learning rate (1e-5;1e-4) - Optimizer: ["AdamW", "RAdam"] - Batch size 8 - Video augmentation: rotation, translation - Max epochs 20 - Frames: [16] - Loss: bce_logit_loss | **0.720** |
| **MViT (mvit_v2_s)** using right coronary artery | Videos of the right coronary artery (224x224). - Learning rate (1e-5;1e-3) - Optimizer: ["AdamW", "RAdam"] - Batch size 8 - No video augmentation  - Loss: bce_logit_loss - Max epochs 20 - Frames: [16] | **0.710** |
| **Swind3D: swin3d_s** | Videos of the left and right coronary artery (224x224). - Learning rate (1e-05;2e-04) - Optimizer: ["AdamW", "RAdam"] - Batch size 8 - No image augmentation - Loss: bce_logit_loss - Max epochs 20 - Frames: [24,36] | **0.57** |
| **R2+1D** | - Videos of the left and right coronary artery (224x224).  -Learning rate {5e-3}  -Binary Cross Entropy Loss  -Batch size 20  - Adam optimizer  -Max Epochs 50  -No image augmentations | **0.500** |

**Table 7. DeepRV Model Card**

**Model Details**

**Overview**

DeepRV is an open-weight, video-based deep neural network that automatically assesses right ventricular systolic function (RVSF) from routine coronary angiography videos.

**Model Description**

- **Architecture**: X3D-Medium (3D convolutional neural network)
- **Input**: Coronary angiography videos (left and right coronary arteries)
- **Output**: Binary classification (Normal vs Reduced RVSF)
- **Training Data**: 8,053 angiographic studies from Montreal Heart Institute (2017-2023)
- **Model Weights**: Available at https://github.com/HeartWise-AI/DeepRV

**Development Team**

- Montreal Heart Institute, Université de Montréal
- University of California, San Francisco
- HeartWise.ai

**Intended Use**

**Primary Use Case**

**Research purposes only** - This model is intended for academic research and development of AI methods in cardiology. It is **NOT approved for clinical use**.

**Target Users**

- Researchers in computational cardiology
- Academic institutions
- AI/ML developers in healthcare

**Out-of-Scope Uses**

- Clinical decision-making
- Patient diagnosis or treatment
- Commercial healthcare applications
- Any use without appropriate research ethics approval

**Training Data**

**Dataset Characteristics**

- **Size**: 82,120 videos from 6,923 patients
- **Demographics**: Mean age 67.4 years, 71% male, 29% female
- **Ground Truth**: Transthoracic echocardiography (TAPSE measurements)
- **Prevalence**: 10.3% reduced RVSF

**Performance Metrics**

**Overall Performance**

- **AUROC**: 0.80 (95% CI: 0.76-0.84) - Internal test set
- **AUROC**: 0.75 (95% CI: 0.72-0.77) - External validation (UCSF)
- **Sensitivity**: 70.5%
- **Specificity**: 78.5%
- **NPV**: 95.8%
- **PPV**: 27.7%

**Performance by Demographics**

**Age Groups**

| **Age Group** | **AUROC (95% CI)** |
| --- | --- |
| <50 years | 0.73 (0.55-0.88) |
| 50-64 years | 0.84 (0.77-0.90) |
| 65-74 years | 0.79 (0.73-0.86) |
| ≥75 years | 0.79 (0.71-0.85) |

**Sex**

| **Sex** | **AUROC (95% CI)** |
| --- | --- |
| Male | 0.80 (0.75-0.84) |
| Female | 0.81 (0.73-0.87) |

**Performance by Clinical Context**

- **STEMI**: AUROC 0.65 (0.52-0.79)
- **NSTEMI/UA**: AUROC 0.76 (0.64-0.86)
- **Heart Failure**: AUROC 0.85 (0.75-0.92)

**Limitations**

**Technical Limitations**

- Lower performance in STEMI patients
- Requires minimum 7 frames at 7–15 fps
- Performance varies with coronary occlusion status
- Limited data for cardiogenic shock patients (n=4)

**Demographic Limitations**

- **No ethnicity/race data collected** - Performance across racial/ethnic groups unknown
- Limited representation of patients <50 years
- Single-center development (Montreal Heart Institute)
- May not generalize to all populations

**Clinical Limitations**

- Temporal mismatch between angiography and echocardiography (up to 5 days)
- Not validated for real-time clinical decisions
- Lower PPV (28%) limits use as standalone diagnostic tool

**Ethical Considerations**

**Fairness**

- Model performance not evaluated across racial/ethnic groups due to absence of this data
- Potential for disparate impact on underrepresented populations
- Users should evaluate performance in their specific populations before research use

**Privacy**

- Model trained on de-identified data
- No patient identifiers in model weights

**Usage Requirements**

**Open-Source Requirement**

**Any work using DeepRV must be open source**. This includes:

- Publishing source code
- Sharing derived models
- Making research findings publicly available

**Citation**

If using DeepRV, please cite the manuscript.

**Access**

- Model weights: https://github.com/HeartWise-AI/DeepRV
- Docker container available for reproducibility
- Research collaboration: robert.avram.md@gmail.com

**Regulatory Status**

- **Not FDA approved**
- **Not CE marked**
- **For research use only**

**Version**

- Current version: 1.0
- Release date: 2025
- Last updated: January 2025

**Disclaimer**

This model is provided "as is" for research purposes only. The developers make no warranties regarding its performance, safety, or suitability for any clinical purpose. Users assume all risks associated with its use.
